# C57BL/6 life span study: age-related declines in muscle power production and contractile velocity

**DOI:** 10.1007/s11357-015-9773-1

**Published:** 2015-04-17

**Authors:** Ted G. Graber, Jong-Hee Kim, Robert W. Grange, Linda K. McLoon, LaDora V. Thompson

**Affiliations:** Program in Physical Therapy, Department of Physical Medicine and Rehabilitation, University of Minnesota Medical School, Rm 366A Children’s Rehab Center, 426 Church Street SE, Minneapolis, MN 55455 USA; Department of Human Nutrition, Foods, and Exercise, Virginia Tech University, Blacksburg, VA 24061 USA; Department of Ophthalmology and Visual Neurosciences, University of Minnesota Medical School, Minneapolis, MN 55455 USA; Department of Physical Education, College of Performing Arts and Sport, Hanyang University, Seoul, Korea

**Keywords:** Mice, Sarcopenia, Muscle, Contractile physiology, Power, Velocity

## Abstract

Quantification of key outcome measures in animal models of aging is an important step preceding intervention testing. One such measurement, skeletal muscle power generation (force * velocity), is critical for dynamic movement. Prior research focused on maximum power (*P*_max_), which occurs around 30–40 % of maximum load. However, movement occurs over the entire load range. Thus, the primary purpose of this study was to determine the effect of age on power generation during concentric contractions in the extensor digitorum longus (EDL) and soleus muscles over the load range from 10 to 90 % of peak isometric tetanic force (*P*_0_). Adult, old, and elderly male C57BL/6 mice were examined for contractile function (6–7 months old, 100 % survival; ~24 months, 75 %; and ~28 months, <50 %, respectively). Mice at other ages (5–32 months) were also tested for regression modeling. We hypothesized and found that power decreased with age not only at *P*_max_ but also over the load range. Importantly, we found greater age-associated deficits in both power and velocity when the muscles were contracting concentrically against heavy loads (>50 % *P*_0_). The shape of the force-velocity curve also changed with age (*a*/*P*_0_ increased). In addition, there were prolonged contraction times to maximum force and shifts in the distribution of the myosin light and heavy chain isoforms in the EDL. The results demonstrate that age-associated difficulty in movement during challenging tasks is likely due, in addition to overall reduced force output, to an accelerated deterioration of power production and contractile velocity under heavily loaded conditions.

**Electronic supplementary material:**

The online version of this article (doi:10.1007/s11357-015-9773-1) contains supplementary material, which is available to authorized users.

## Introduction

One major health challenge facing our aging population is sarcopenia, the age-related loss of muscle mass and strength (Thompson 2009). Sarcopenia not only decreases quality of life and makes activities of daily living more difficult, but it leads to loss of independence and is a major component of frailty (Landi et al. 2013; Fielding et al. 2011). Frailty is a syndrome characterized by low health reserve, decreased strength, generally poor prognosis for outcomes after medical procedures, and increased mortality (Macklai et al. 2013). There is no cure for sarcopenia, though exercise (resistance training in particular) does show promise as a treatment because it increases muscle mass, strength, and function (Chodzko-Zajko et al. 2009; Fiatarone et al. 1990; Evans 1998). Movement requires skeletal muscle power generation. Skeletal muscle power is the product of two contractile parameters: force and velocity.

Research in mice to determine contractile properties in vitro has traditionally focused on *P*_0_ (maximum isometric force production), *V*_max_ (a nonphysiological measure of unloaded velocity derived from curve-fitting force-velocity data with the Hill equation), and *P*_max_ (maximum power, generally achieved at 30–40 % *P*_0_). *P*_0_ and *P*_max_ decline with age (Lynch et al. 2001; Brooks and Faulkner 1988a, b), whereas an age-associated decrease in *V*_max_ has not been reported (Brooks and Faulkner 1988a). Similar to mice, *P*_max_ and maximum torque (strength) decline with age in humans, when measured as a joint movement (Reid et al. 2014; Narici et al. 1991). Plantar flexor motion demonstrates a decrease in both power and angular velocity with age in humans (Clemencon et al. 2008). Therefore, it has been suggested that the loss of power production in the elderly is due to declines in both angular velocity and torque (Power et al. 2013; Dalton et al. 2014). Human studies are necessarily performed in vivo, but in the mouse model, it is possible to reduce the complexity of the in vivo system to a single muscle using the in vitro method.

*P*_max_ occurs within a narrow range of % *P*_0_, which does not encompass all types of dynamic movement. The force-power curve of a muscle is a hyperbola that peaks at 30–40 % *P*_0_ (*P*_max_). Zero power is produced at zero load (0 % *P*_0_), a hypothetical value because a muscle is always physiologically loaded. Power production is also 0 at 100 % of *P*_0_ (isometric contraction). Movement and physical performance require power production, and in the elderly, a decline in power production represents an important indicator of physical function (Reid and Fielding 2012). Many functional activities require power production spanning the breadth of the force-power curve. Examples of human movement at the low range of the force-power curve include picking up a cup of coffee or folding a shirt, whereas at the high end activities such as opening a tightly sealed jar or arresting a fall require power production at forces closer to maximal *P*_0_.

The emphasis on power production over the full force-power curve, by determining contractile velocity from 10–90 % *P*_0_, is an important expansion of the previously published in vitro contractility literature. For instance, Lynch et al. 2001 determined *P*_max_ using *V*_opt_ (maximum contractile velocity that produces the maximum power output) and Brooks and Faulkner (1988a, b) determined contractile velocity at <50 % *P*_0_ in order to determine *V*_max_. Therefore, the primary purpose of the current study was to investigate power production in concentric muscle contractions against loads from light (10 % *P*_0_) to heavy (90 % *P*_0_) across the life span of the male C57BL/6 mouse, a commonly used aging model. The overall hypothesis was that power would decrease over the entire load range with age, with an age-associated loss of velocity and an overall reduction in force both contributing to the power reduction. Characterizing the effects of age on skeletal muscle power production over the entire load range and over the life span of the mouse will provide the foundation to design interventions for preclinical sarcopenia models.

To test our hypothesis, the maximum force (*P*_0_) and the force-velocity relationship of both the soleus (SOL) and the extensor digitorum longus (EDL) were determined in vitro. The SOL is comprised of ~40 % type 1 and 60 % type 2 muscle fibers (Burkholder et al. 1994). The EDL is comprised of mainly type 2 muscle fibers (Burkholder et al. 1994). The force-power curve was then derived from the force-velocity relation. In addition, we tested for an age-related change in the distribution of myosin isoforms (myosin heavy chains and myosin light chains), since these proteins are major determinants of fiber contractile velocity (Moss et al. 1995; Larsson et al. 1997).

## Methods

### Animals

Male C57BL/6 mice (*n* = 80) from three age groups were analyzed [adult (A, *n* = 15; 5–7 months old, 100 % survival), old (O, *n* = 14; 22–26 months, ~75 % survival), and elderly (E, *n* = 24; 28+ months, <50 % survival)] with additional mice outside of the three age groups used for regression analysis. All animals were from 5 to 32 months of age. The mice were obtained from the NIA aging colony at Charles River Laboratories (Charles River, MD). Some of these animals (*n* = 50) were used in a previous study (Graber et al. 2013), where we reported peak tetanic force, rotarod, and grip test outcome measures. Mouse husbandry was performed by core staff in a specific pathogen-free facility. IACUC-approved standard operating procedures were used to assure humane and ethical treatment. Body and muscle mass were measured just prior to the physiology experiment, and body mass was also assessed 1 week after the mice arrived.

### Whole muscle physiology

Tissue handling, equipment and settings, determination of optimal length, peak twitch force, maximum tetanic force, and the force frequency curve are described in detail elsewhere (Graber et al. 2013). In brief, while under deep anesthesia, the SOL and EDL muscles were carefully removed from the animal and weighed. The muscles were tied with #4 silk suture just above their origin and insertion myotendinous junctions and were attached vertically between platinum electrodes to a force transducer (300B, Aurora Scientific Inc. (ASI), Aurora, Ontario, Canada) and a static clamp in a tissue chamber. The muscle was then submerged in Krebs-Ringer buffer in a vertical oxygenated bath (maintained at 25 °C; Graber et al. 2013). The muscle was stimulated (using 701B Stimulator, software controlled with Dynamic Muscle Control version 3.2; ASI) with a single pulse of 500 μs, at 30 V and 1 A, while length was increased until optimal length (*L*_0_, measured with a caliper) was achieved—the point at which the peak twitch force (*P*_t_) was determined. The muscles were then held at *L*_0_ and stimulated at various frequencies, between 10 and 180 Hz, depending on muscle type to determine the peak tetanic force (*P*_0_). Physiological cross-sectional area (PCSA) was determined using the average density of skeletal muscle by the formula: PCSA (cm^2^) = Muscle mass (g) / [*L*_0_ (cm) * 1.06 (g/cm^3^)] (Mendez and Keys 1960).

### Force-velocity curve

Using *P*_0_ as the reference force, maintaining *L*_0_ (optimal fiber length), and stimulating at the frequency where *P*_0_ was found, the force-velocity curve was determined by using the load-clamp technique to measure velocity at various percentages of *P*_0_ (10–90 %). Specifically, the load-clamp technique sets the force transducer arm to resist at the % *P*_0_, but the arm can travel freely once the muscle produces force that exceeds the load. The action can be thought of as moving against resistance, as in weight lifting. The muscle was maximally stimulated for 500 ms. The distance the arm moved over the time of stimulation was used to compute the maximum velocity at % *P*_0_ by finding where the first derivative of the time-distance curve equaled 0. The absolute measurement of velocity was in millimeters per second (mm/s), which was converted to a normalized measurement of fiber lengths per second (fl/s) using the established ratio values of intact fiber length to muscle length: 0.44 (EDL, adult), 0.45 (EDL, old and elderly), and 0.69 (SOL) (Brooks and Faulkner 1988a). The force-velocity curve was constructed, and the values entered into a custom MATLAB (Natick, MA) program. The custom MATLAB program fits the data to a curve using the Hill equation (Woledge et al. 1985), (*V* + *b*)(*P*/*P*_0_ ± *a*/*P*_0_) = *b*(1 + *a*/*P*_0_), to extrapolate the maximum unloaded velocity (*V*_max_) and also plots a force-power curve (by fitting the data to a 5th degree polynomial curve) to determine both the peak power (*P*_max_) and the percentage of *P*_0_ at *P*_max_ (% *P*_max_).

### Force-power curve

The power at the % *P*_0_ loads (force-power curve) was determined at the measured percentages of *P*_0_ by multiplying force at % *P*_0_ and the maximum velocity at the % *P*_0_, determined as described above. The *P*_max_ and % *P*_max_ were determined from the MATLAB output.

### Myosin light chain and heavy chain isoforms

This procedure has been previously described (Thompson et al. 2006). In brief, proteins from EDL and SOL samples, from whole muscle homogenates—made from muscles that were stored in skinning solution (which permeabilizes the individual muscle fibers), were separated on large format electrophoresis gels at 12 % acrylamide for light chain analysis and 5 % for heavy chain analysis and then silver stained. Proteins were positively identified by using tandem mass spectrometry/mass spectrometry on a Thermo Scientific LTQ Orbitrap to identify the proteins on the standards. The standards were made from rat whole muscle homogenates (TA, SOL, and diaphragm). The gels were imaged using a Bio-Rad Gel-Doc XRS Imager (Hercules, CA), and the relative protein abundance was calculated with densitometry using QuantityOne software (Bio-Rad).

### Statistics

One-way ANOVA (Tukey-Kramer honestly significant difference (HSD) post hoc) or Student’s *t* test was used to detect differences between means, as appropriate. Statistical significance was at *p* < 0.05. Both simple and multiple linear regressions were analyzed to examine relationships between variables. A general linear model was used to compare slopes of regression lines. Principal component analysis using a promax rotation was used to determine the most significant variables for multiple regressions. We used SPSS v. 20 (IBM Corporation, Armonk, NY) for all statistical analyses. Data are presented as means ± standard error (SEM).

## Results

### Force-velocity relationship and *V*_max_

**Fig. 1:**
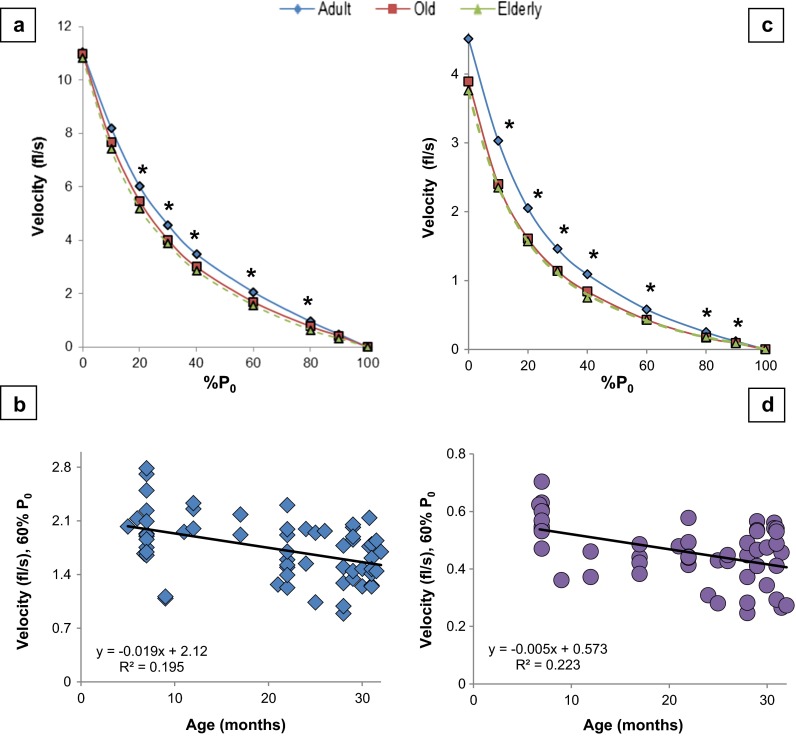
Velocity declines with age. **a** EDL force-velocity curve. **b** Linear regression of EDL velocity at 60 % *P*_0_. See Fig. S1 in the supplement for the other velocities. **c** SOL force-velocity curve. **d** Linear regression of SOL velocity at 60 % *P*_0_. See Fig. S2 in the supplement for the other velocities. See Table 1 and text for post hoc analysis. *Each symbol* in **b** and **d** in the regression graphs represents a measurement from an individual mouse at the given age. Equation: simple linear regression of velocity (*y*) as a function of age (*x*). *p < 0.05, *p* value from one-way ANOVA. *fl/s* fiber length per second, *% P*_*0*_ percentage of maximum isometric force, *Age* age in months

The velocities of contraction under loaded (>0 % *P*_0_) conditions showed age-related declines (Fig. 1 and Table 1). In the elderly mice, the extent of reduction in contractile velocities averaged 21 ± 4 % in the EDL (range of 10–34 %) and 24 ± 2 % in the SOL (range of 16–33 %), with no significant difference in the mean decline between the two muscles [Student’s *t* test comparing % reductions in velocity in elderly mice from 10 to 90 % *P*_0_: EDL and SOL; *t* = −1.1, *p* = 0.290]. In the old mice, EDL velocities at 30–60 % *P*_0_ showed an age-associated reduction compared to the adult. For the old mice, there was slower contraction speed in the SOL compared to the adults from 10 to 80 % *P*_0_. Since the old mice were not significantly different from the elderly in contractile velocity, we report herein, for brevity, only the differences in velocity between adult and elderly mice.

**Table 1:** Power and velocity at percentages of maximum force (% *P*_0_)

Power (mN fl/s)						Velocity (fl/s)				
% *P*_0_	Adult	Old	Elderly	*p* value	%Change	Adult	Old	Elderly	*p* value	%Change
EDL										
0 (*V*_max_)	0	0	0			11.0 ± 0.3	11.0 ± 0.4	10.8 ± 0.3	0.921	−2
10	318 ± 16	272 ± 14	204 ± 11ab	<0.001	−36	8.2 ± 0.2	7.7 ± 0.2	7.4 ± 0.2	0.064	−10
20	466 ± 23	388 ± 19a	289 ± 16ab	<0.001	−38	6.0 ± 0.2	5.5 ± 0.1	5.2 ± 0.2a	0.004	−13
30	531 ± 26	428 ± 21a	322 ± 18ab	<0.001	−39	4.6 ± 0.1	4.0 ± 0.1a	3.9 ± 0.1a	0.001	−15
40	539 ± 29	424 ± 23a	316 ± 18ab	<0.001	−41	3.5 ± 0.1	3.0 ± 0.1a	2.8 ± 0.1a	0.002	−20
60	476 ± 26	359 ± 25a	259 ± 16ab	<0.001	−46	2.1 ± 0.1	1.7 ± 0.1a	1.6 ± 0.1a	<0.001	−24
80	295 ± 26	215 ± 27a	139 ± 12ab	<0.001	−53	0.96 ± 0.08	0.77 ± 0.09	0.63 ± 0.05a	0.004	−34
90	168 ± 21	132 ± 22	78 ± 10a	<0.001	−54	0.48 ± 0.07	0.42 ± 0.06	0.32 ± 0.04	0.064	−34
100	0	0	0			0	0	0		
*P*_max_^a^	550 ± 27	437 ± 17a	330 ± 18ab	<0.001	−40					
% *P*_0_ @ *P*_max_	38 ± 1.1	35 ± 0.9	33 ± 0.6a	<0.001	−13					
*a*/*P*_0_	0.0058	0.0084	0.012ab	<0.001	+106					
Soleus										
0 (*V*_max_)	0	0	0			4.6 ± 0.2	3.9 ± 0.3	3.8 ± 0.2	0.097	−17
10	58 ± 4	49 ± 5	40 ± 3a	0.006	−31	3.1 ± 0.1	2.4 ± 0.1a	2.4 ± 0.1a	0.001	−23
20	78 ± 5	65 ± 6	53 ± 4a	0.003	−32	2.1 ± 0.1	1.6 ± 0.1a	1.6 ± 0.1a	<0.001	−24
30	82 ± 6	69 ± 7	58 ± 4a	0.006	−30	1.5 ± 0.1	1.2 ± 0.05a	1.2 ± 0.07a	0.001	−20
40	81 ± 6	69 ± 6a	51 ± 5a	0.001	−38	1.1 ± 0.04	0.8 ± 0.04a	0.8 ± 0.05a	0.001	−27
60	66 ± 5	51 ± 4	43 ± 3a	0.001	−35	0.6 ± 0.02	0.4 ± 0.02a	0.4 ± 0.02a	<0.001	−33
80	38 ± 4	27 ± 3a	24 ± 2a	0.001	−37	0.3 ± 0.04	0.2 ± 0.02a	0.2 ± 0.01a	0.002	−33
90	23 ± 4	15 ± 2	14 ± 1a	0.020	−39	0.13 ± 0.02	0.09 ± 0.01	0.09 ± 0.01	0.081	−31
100	0	0	0			0	0	0		
*P*_max_^a^	81 ± 6	70 ± 7	58 ± 4a	0.008	−28					
% *P*_0_ @ *P*_max_	31 ± 0.3	29 ± 0.9	29 ± 0.3	0.266	−6					
*a*/*P*_0_	0.021	0.024	0.027	0.067	+29					

*p* value was taken from one-way ANOVA. Note: a = different than adult and b = different than old (*p* < 0.05) (marked only on old and elderly)

*V*_*max*_ maximum unloaded velocity, *P*_*max*_ maximum power, *% P*_*0*_ *@ P*_*max*_ percentage of maximum force where *P*_max_ was produced, *mN fl/s* millinewtons multiplied by fiber lengths per second, *%Change* change from adult to elderly

^a^ *P*_max_ values from MATLAB data curve-fit, force-power curve derived from the maximum instantaneous velocity (at % *P*_0_) multiplied by force (at the % *P*_0_). In the adult SOL, the derived *P*_max_ (81 ± 6) is not statistically different than the derived value for power at 30 % *P*_0_ (82 ± 6)

#### Elderly EDL

The contractile velocities of the elderly, between 20 and 80 % *P*_0_, were significantly slower than the adult EDL group (Fig. 1a, b and Table 1). Specifically, at loads <50 % *P*_0_ (10, 20, 30, and 40 %), the velocities of the elderly group were reduced by 10 (not significant), 13, 15, and 20 % (*p* values = 0.051, 0.004, 0.001, and 0.002), respectively. In contrast, at loads of 60, 80, and 90 (not significant), the velocities were reduced by 24, 34, and 34 % (*p* values = <0.001, 0.004, and 0.064), respectively.

**Fig. 2:**
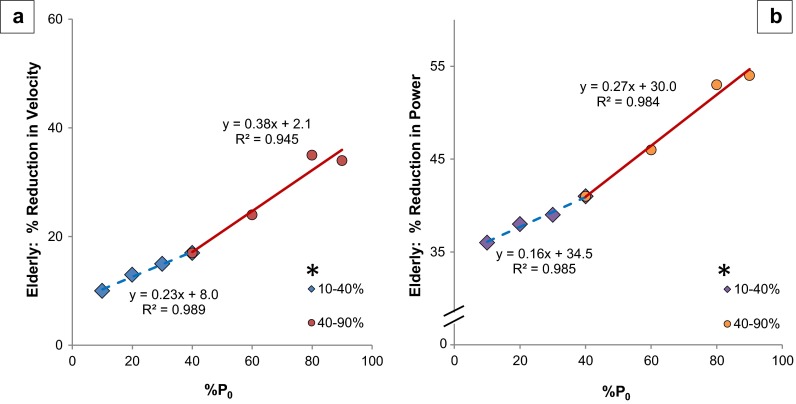
EDL velocity and power at heavier loads show increased age-related rate of decline. **a** Velocity. **b** Power equation: simple linear regression of % reduction in velocity and power of elderly mice (in comparison to adult mice) as a function of load (% *P*_0_). Light loads (10–40 % *P*_0_) are represented by *diamonds with a dashed regression line* and heavy loads (40–90 % *P*_0_) are represented by *circles and a solid regression line*; 40 % *P*_0_ is the inflection point indicating where the slope changes, and as such, the point is included in both sections of the graph. * = slope of line significantly different, *p* = 0.003 and *p* < 0.001 for velocity and power, respectively

While *V*_max_ (unloaded velocity) did not change, age exerted a greater effect on EDL contractile velocity as the muscles contracted under increased loads (simple regression of the percent reduction and % *P*_0_: %Velocity = 0.33 (% *P*_0_) + 5.7, *R* = 0.985) (data not shown). The mean velocity percentage (average percent change at all measured loads) was reduced twofold at heavier loads (40–90 % *P*_0_) to 28 %, in comparison to lighter loads (reduced 14 % from 10 to 40 % *P*_0_) (Table 1 and Fig. 2a). There was an additional age-related decline of 65 % (slope of simple linear regression lines) at the heavier loads in comparison to the lighter loads [from separate linear regressions: %Velocity @ 10–40 % *P*_0_ = 0.23 (% *P*_0_) + 8.0, *R* = 0.994; %Velocity @ 40–80 % *P*_0_ = 0.38 (% *P*_0_) + 2.1, *R* = 0.972]. The slopes of these regression lines were different (*p* = 0.003, from the general linear model) (Fig. 2).

#### Elderly SOL

The SOL *V*_max_ was not significantly different in the elderly (−17 %, ANOVA *p* = 0.072, Tukey’s HSD *p* = 0.061). However, the contraction velocity was significantly reduced by 23, 24, and 20 %, at loads of 10, 20, and 30 % *P*_0_, respectively. At the higher loads of 40, 60, 80, and 90 % *P*_0_, the elderly mice contracted slower than the adults—27, 33, 33, and 31 %, respectively (90 % *P*_0_ not significant, *p* = 0.081) (Table 1 and Fig. 1c, d).

Similar to the EDL, the effect of age became more pronounced at higher loads (simple linear regression of the percent reduction and 0–90 % *P*_0_: %Velocity = 0.23 (% *P*_0_) + 16.5, *R* = 0.946). The mean percent reduction in contraction velocity was 19 % between 0 and 30 % *P*_0_, but was 30 % from 40 to 90 % *P*_0_. When analyzing the regressions of the no/low load (0–40 % *P*_0_) and higher load (40–90 % *P*_0_) separately, the slopes of the lines were similar (suggesting a similar rate of effect), and *R* values showed moderate correlation: %Velocity @ 0–40 % *P*_0_ = 0.13 (% *P*_0_) + 17.3, *R* = 0.757; %Velocity @ 40–90 % *P*_0_ = 0.12 (% *P*_0_) + 16.5, *R* = 0.567.

#### Life span—age and velocities

In order to assess the effect of age, from 5 to 32 months, on velocity, we examined linear regressions of age with velocity at the various % *P*_0_. EDL showed a significant negative correlation between age and velocity under loads from 20 to 90 % *P*_0_, whereas the SOL was significant at all loads from 10 to 90 % *P*_0_. All the linear regression graphs with equations are found in the Online Resource 2 Supplemental Figs. S1 and S2 [future references will use the convention “Fig. Sx,” representing the figure number in Online Resource 2 Supplemental figures (S1–S7)].

### Shape of force-velocity curve, *a*/*P*_0_

**Fig. 3:**
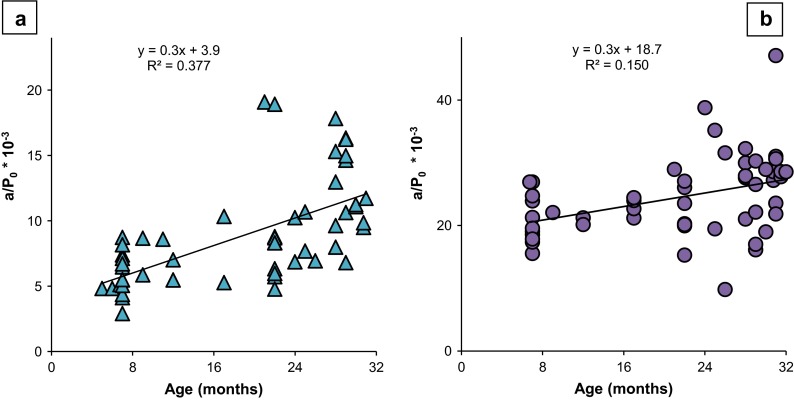
Force-velocity curve changes with age: *a*/*P*_0_ increases with age. **a** EDL. **b** SOL. Equation is a simple linear regression of *a*/*P*_0_, which describes the shape of the force-velocity curve, with respect to age of the mouse in months. *Each symbol* represents the *a*/*P*_0_ of one mouse

To determine if there was a difference in the shape of the EDL and SOL force-velocity curves, the *a*/*P*_0_ value was evaluated (*a*: constant from the Hill equation, *a*/*P*_0_: describes the shape of the curve, see “Methods”). There was a downward and leftward shift with advancing age in the EDL (one-way ANOVA: *F* = 19.1, *p* < 0.001; Tukey’s HSD: adult vs. elderly *p* < 0.001; old vs. elderly *p* = 0.005) (Table 1). In contrast, the SOL curves did not exhibit a significant shift with age between groups (one-way ANOVA: *F* = 3.401, *p* = 0.067) (Table 1). Over the life span, however, linear regressions showed an increase in *a*/*P*_0_ with age in both the EDL and SOL, thus demonstrating that the force-velocity curve is altered with age (Fig. 3a, b).

### Force-power relationship

In order to determine the influence of age on power production, we constructed and analyzed force-power curves by multiplying the force produced and the velocity of contraction for both the SOL and EDL in the three age groups between 0 and 100 % *P*_0_ and then examined regressions of age (5–32 months) and power at all % *P*_0_.

**Fig. 4:**
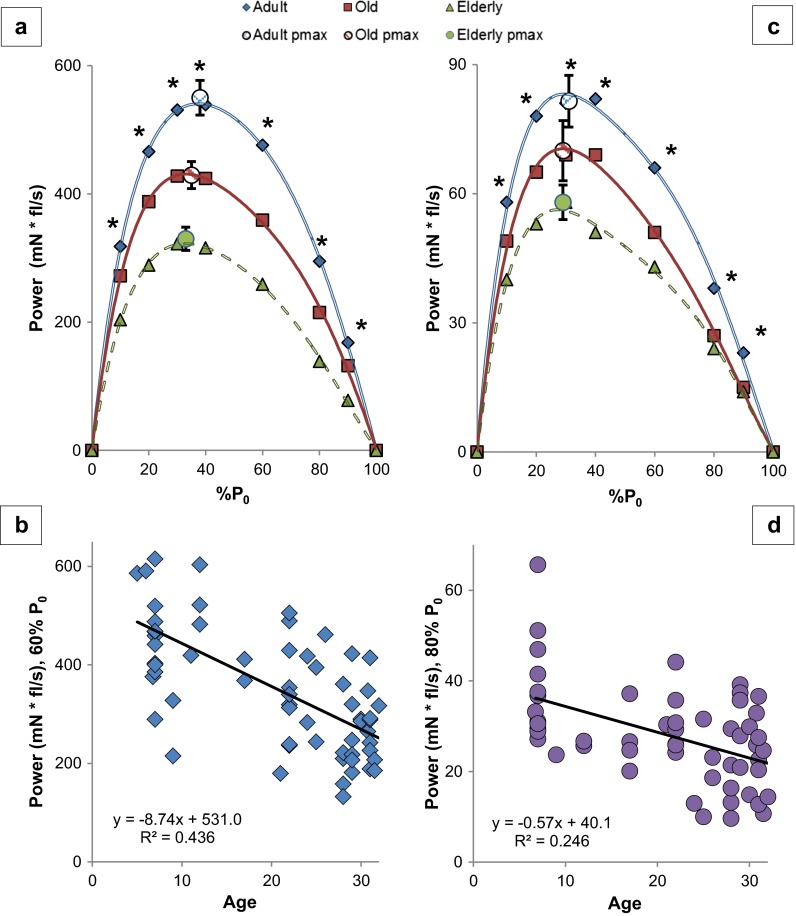
Power declines with age. **a** EDL. **b** Linear regression of EDL power at 60 % *P*_0_. See Fig. S3 in the supplement for the other regressions. **c** SOL. **d** Linear regression of SOL power at 80 % *P*_0_. See Fig. S4 in the supplement for the other regressions. See Table 1 and text for post hoc analysis*. Each symbol* in **b** and **d** in the regression graphs represents a measurement from an individual mouse at the given age. Equation: simple linear regression of power (*y*) as a function of age (*x*). *p < 0.05, *p* value from one-way ANOVA. *mN fl/s* millinewtons multiplied by fiber lengths per second, *% P*_*0*_ percentage of maximum isometric force, *Age* age in months

Power production declined with age (Table 1, Fig. 4, Figs. S3 and S4). In the elderly mice, the reduction in power averaged 43 ± 3 % in the EDL (range of 33–54 %) and 35 ± 3 % in the SOL (range of 31–39 %), across the loads of 10–90 % *P*_0_ but there was no significant difference in percentage power reduction between the two muscles [Student’s *t* test: *t* = 2.15, *p* = 0.052]. In the old mice, the EDL had an overall power reduction of 20 % (mean of all loads) and the SOL averaged 32 % over the higher loads of 60–80 % *P*_0_. However, in the old SOL, the decline observed from 10 to 40 % *P*_0_ was not significant, so we did not compare the old EDL to the old SOL with respect to the average percent change. Elderly mice had significantly reduced power at 10–80 % *P*_0_ (at 90 % *P*_0_, *p* = 0.052) in the EDL, averaging 29 % and ranging from 25 to 41 %, compared to old EDL. In the SOL, the old and elderly power production was not significantly different. In the interest of brevity, we will discuss in depth only the specific differences between the adult and elderly mice.

**Fig. 5:**
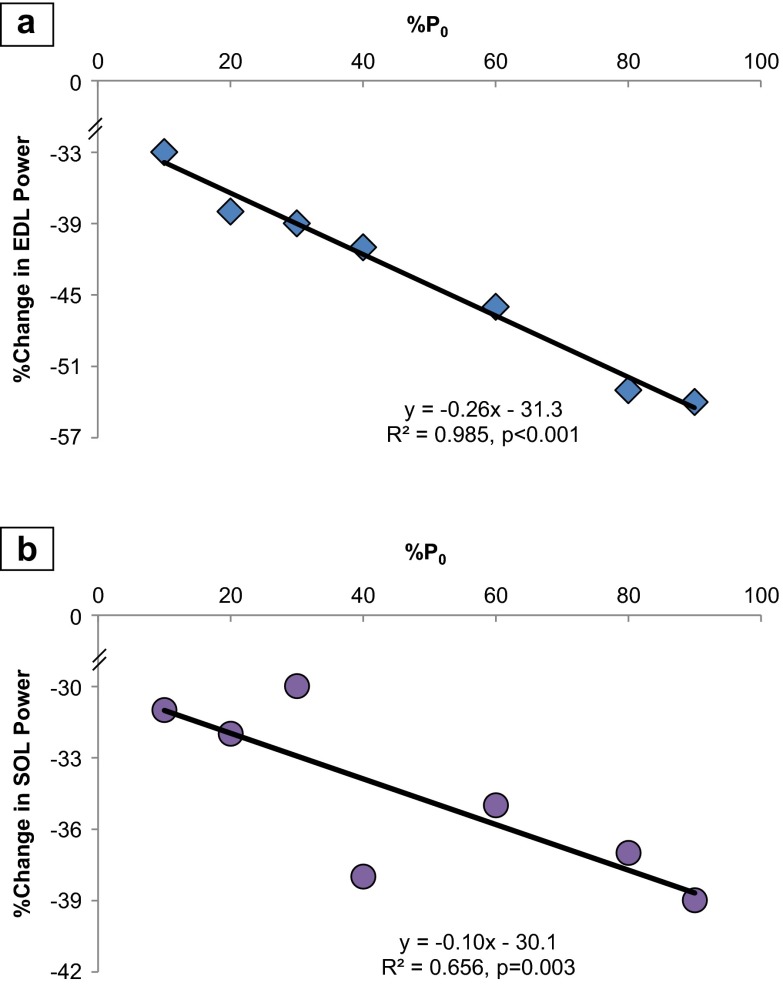
Percent reduction in power is correlated with increases in % *P*_0_, comparing the elderly to adult. **a** EDL: There was a −0.26 % reduction in power per unit increase in load (% *P*_0_, percentage of maximum force at which power was derived) (*R* = 0.993). **b** SOL: There was a percentage decrease of −0.10 (*R* = 0.810) in power, per unit increase in % *P*_0_. *Each symbol* represents the difference between the mean power production of the entire adult group compared to the entire elderly group. Equation: simple linear regression of percent change in power from adult to elderly (*y*) as a function of % *P*_0_

The percent reduction in power by the EDL per unit increase in load (% *P*_0_) had a linear relationship that amounted to −0.26 % (*R* = 0.993, *p* < 0.001). Likewise, the SOL had a percentage decrease of −0.10 (*R* = 0.810, *p* = 0.003) in power, per unit increase in % *P*_0_ (Fig. 5a, b).

#### Elderly EDL

As noted above, power production was significantly reduced at loads between 10 and 90 % *P*_0_ in the elderly EDL group. At loads <50 % *P*_0_ (10, 20, 30, and 40), the power was reduced by 36, 38, 39, and 41 %, respectively (Table 1, Figs. 2b, 4a, b, and 5a). In contrast, at loads of 60, 80, and 90 %, power was reduced by 46, 53, and 54 %, respectively. Age exerted a greater effect on EDL power production as the loads increased (simple regression of the percent reduction and % *P*_0_: %Power reduction = −0.26 (% *P*_0_) − 31.3, *R* = 0.991, *p* < 0.001) (Fig. 5a). Power was reduced 31 % more at heavier loads (overall 51 % mean reduction from 60 to 90 % *P*_0_) compared to lighter loads (overall 39 % mean reduction from 10 to 40 % *P*_0_). The age-related decline was 57 % more (comparing slopes of regression lines) in the heavier loads compared to the lighter loads [from separate linear regressions: %Power @ 10–40 % *P*_0_ = 0.16 (% *P*_0_) + 34.5, *R* = 0.992; %Power @ 40–90 % *P*_0_ = 0.28 (% *P*_0_) + 30.0, *R* = 0.992] (Fig. 2b, Fig. S4). The slopes of the regression lines were different (*p* < 0.001, from the general linear model).

#### Elderly SOL

Specifically, at the lighter loads of 10, 20, and 30 % *P*_0_, power was reduced by 31, 32, and 30 %, respectively. At the higher loads of 40, 60, 80, and 90 % *P*_0_, power was reduced by 38, 35, 37, and 39 %, respectively (Table 1, Figs. 4c, d and 5b). As in the EDL, the effect of age became more pronounced at higher loads (simple linear regression of the percent reduction and 0–90 % *P*_0_: %Power = −0.10 (% *P*_0_) − 30.1, *R* = 0.656, *p* = 0.003) (Fig. 5b). The mean percent reduction in power was 31 % from 10 to 30 % *P*_0_, but was 37 % from 40 to 90 % *P*_0_. However, there was no indication of an increase in the rate of effect [from separate linear regressions of the no/low load (0–40 % *P*_0_) and higher load (40–90 % *P*_0_), the slopes of the lines were similar, and *R* values showed a moderately strong correlation: %Power @ 0–40 % *P*_0_ = 0.23 (% *P*_0_) + 25.5, *R* = 0.769; %Power @ 40–90 % *P*_0_ = 0.21 (% *P*_0_) + 25.9, *R* = 0.819] (data not shown).

#### Life span—age and power

To determine correlation and determine predictive equations, we examined linear regressions of age (from 5 to 32 months) with power at the various % *P*_0_. Both the EDL and SOL showed significant negative correlations between age and power under all loads (Figs. S3 and S4).

#### *P*_max_ and % *P*_0_ @ *P*_max_

The EDL *P*_max_ of the elderly and old mice was reduced 40 % (*p* < 0.001) and 21 % (*p* = 0.001), respectively, when compared to the adults (Table 1). EDL *P*_max_ also declined 24 % between old and elderly mice (*p* = 0.015). Likewise, a simple linear regression of the EDL *P*_max_ with age revealed a significant decline [(*R* = −0.700, *r*^2^ = 0.489, *p* < 0.001) with the equation: *P*_max_ (mN fl/s) = 600.8 − 9.1 * age (months)] (Fig. S3). The EDL % *P*_0_ @ *P*_max_ was reduced 13 % (*p* < 0.001) in the elderly (33 %) and 8 % (not significant, *p* = 0.069) in the old (35 %) compared to the adult (38 %).

The SOL *P*_max_ reduced with age, 28 % (*p* = 0.009) in the elderly with no reduction in the old group (Table 1). Regression of the SOL *P*_max_ with age, however, revealed a significant decline over the life span (*R* = −0.301, *r*^2^ = 0.091, *p* = 0.052) with the equation: *P*_max_ (mN fl/s) = 79.5 − 0.7 * age (months) (Fig. S4). SOL % *P*_0_ @ *P*_max_ of the adult and elderly mice was not different (−6 %).

#### Relationships between age, force, velocity, and maximum power

In order to evaluate the relationship between age, velocity, force, and *P*_max_, we examined simple and multiple linear regressions to determine how much variability is explained by the velocity and force components of power (equations and description of the procedures are found in the Online Resource 1 Appendix). In brief, in the EDL, *P*_0_ explained 58 % of the individual variability in *P*_max_ (*P*_0_ + age, 66 %), while velocity of contraction explained 23 % (velocity + age, 49 %). Both velocity and *P*_0_ combined to account for 73 % (with age, 75 %). In the SOL, the *P*_0_ could explain 59 % of the individual variability in *P*_max_ (*P*_0_ + age, not significantly different), velocity of contraction 50 % (velocity + age, no difference), and *P*_0_ and velocity combined, 83 % (with age, 84 %) (Fig. S5).

### Myosin heavy and light chain composition

In the EDL, the percentage of myosin heavy chain (MHC) isoform 2a/x was increased, with the adults at 14 % and the older mice at 20 % of the total myosin, and the 2b isoform (adult 86 % and older 80 %) decreased by 6 % with age, adults at 86 % and old mice at 80 % (bundle electrophoresis) (Student’s *t* test, *p* = 0.009 and 0.010, respectively) (Fig. S6). There was also a 21 % decrease in the percentage of myosin light chain 3f in the EDL of older mice (combined old and elderly, *n* = 23, mean age = 28) when compared to adult (*n* = 19, mean age = 7.5) (Fig. S7, Student’s *t* test: *p* = 0.030). There was no difference in MHC 2a/x expression in the SOL (61.8 % in adult and 61.0 % in older mice, Student’s *t* test: *p* = 0.670) or in MLC3f expression (11.0 % in adult and 9.5 % in older mice, Student’s *t* test: *p* = 0.200) (Figs. S6 and S7).

### Animal characteristics

Table 2 summarizes the animal characteristics critical to interpreting the effect of age on muscle contractile velocity and power production. The body mass of the mice in the older group (75 % survival) was heavier than both the adult (100 % survival) and elderly (<50 % survival) groups. Specifically, the body mass of the mice in the EDL old group was 17 % heavier compared to the adults (*p* = 0.008) and 13 % greater than the elderly EDL group (*p* = 0.018). In the SOL group, the old mice were 33 % heavier when compared to the adult (*p* = 0.001) and 27 % more than the elderly (*p* = 0.001) (ANOVA, with Tukey’s HSD post hoc).

**Table 2:** Animal characteristics for EDL (*n* = 53) and SOL (*n* = 47) groups. Adult mice (EDL *n* = 15, SOL *n* = 12) were used as the reference group to the old (EDL *n* = 14, SOL *n* = 12) and elderly (EDL *n* = 24, SOL = 23). *p* value was taken from one-way ANOVA (Tukey’s HSD post hoc test)

	Adult (5–7 months)	Old (22–26 months)	Elderly (27–32 months)	*p* value
EDL				
Mean age (months)	6.8 ± 0.1	23.0 ± 0.4	29.9 ± 0.3	
Body mass (g)	31.7 ± 0.8	37.1 ± 1.6a	32.7 ± 0.7	0.006
Mass (mg)	15.4 ± 3.2	13.4 ± 1.8	11.5 ± 2.3a	<0.001
PCSA (mm^2^)	1.23 ± 0.29	1.02 ± 0.14a	0.9 ± 0.22a	<0.001
*P*_0_ (mN)	388 ± 17	356 ± 16a	279 ± 13a, b	<0.001
SOL				
Mean age (months)	7.0 ± 0.02	23.3 ± 0.5	29.9 ± 0.3	
Body mass (g)	31.1 ± 0.8	41.5 ± 3.0a	32.7 ± 0.7b	<0.001
Mass (mg)	14.0 ± 1.4	12.2 ± 0.4	11.9 ± 0.6	0.197
PCSA (mm^2^)	1.16 ± 0.11	1.04 ± 0.04	1.03 ± 0.06	0.431
*P*_0_ (mN)	187 ± 10	195 ± 13	166 ± 7	0.059

Note: a = different than adult and b = different than old (*p* < 0.05) (marked only on old and elderly)

*PCSA* physiological cross-sectional area of muscle, *P*_*0*_ maximum tetanic contractile force, *mN* millinewtons

EDL muscle mass and size decreased with age. EDL muscles in the elderly group had a 25 % reduction in muscle mass (*p* < 0.001) and a 27 % reduction of the PCSA (*p* < 0.001) when compared to the EDL muscles in the adult mice. EDL muscle mass in the old group was not significantly different (−13 %, *p* = 0.085), but there was a 17 % reduction in the PCSA (*p* = 0.039) compared to the adult mice (ANOVA, with Tukey’s HSD post hoc).

When muscle mass was normalized to body mass (mg of muscle mass/grams of body mass, or mg/gbm), the reduction was confirmed. The old EDL (0.37 ± 0.01 mg/gbm) were 25 % smaller, and the elderly (0.35 ± 0.01 mg/gbm) were 28 % smaller (ANOVA *p* < 0.001; Tukey’s HSD *p* = <0.001 for both).

In contrast to the EDL, the SOL muscle mass and PCSA did not show the age-related decrease. However, the normalized muscle mass revealed an age-related decline such that the SOL muscles from the old mice (0.31 ± 0.03 mg/gbm) were 30 % lighter, although muscles from the elderly mice (0.36 ± 0.01 mg/gbm) were not significantly different (−29 %) when compared to the adult group (ANOVA *p* = 0.012; Tukey’s HSD *p* = 0.009 and 0.077, respectively).

### Force production declined with age

With age, the *P*_0_ of the EDL was reduced (Table 2). The elderly produced 28 % less force (*p* < 0.001) and old mice 22 % less (*p* = 0.003), in comparison to the adult. EDL *P*_0_ normalized to muscle cross-sectional area was not different. The normalized EDL *P*_0_ (to body mass) was lower in both the elderly and old mice compared to adult by 29 % (*p* < 0.001) and 20 % (*p* < 0.001), respectively. In contrast, the SOL did not show a significant decline in *P*_0_ (*p* = 0.057). SOL also did not decline in *P*_0_ normalized to muscle cross-sectional area. However, after normalizing SOL *P*_0_ to body mass, there was an age-related decline. The old mice (4.8 ± 0.43 mN/gbm) lost 22 % of normalized force in comparison to the adults (6.1 ± 0.40 mN/gbm), but the elderly (5.1 ± 0.23 mN/gbm) were not significantly different from the adults (ANOVA *p* = 0.042; Tukey’s HSD: old *p* = 0.043 and elderly *p* = 0.103).

To determine the effects of age on EDL *P*_0_ over the life span (5–32 months of age), a simple linear regression of the *P*_0_ with age revealed that age explained 30 % of the variation in *P*_0_ [*R* = 0.550, *r*^2^ = 0.302, *p* < 0.001; equation: *P*_0_ (mN) = 425.99 (mN) − 4.45 * age (months)]. This relationship of EDL and age was similar to the results we found previously (Graber et al. 2013). In contrast, neither the simple regression with age of the SOL *P*_0_ nor the normalized SOL *P*_0_ was different (*p* = 0.121 and 0.140, respectively).

**Fig. 6:**
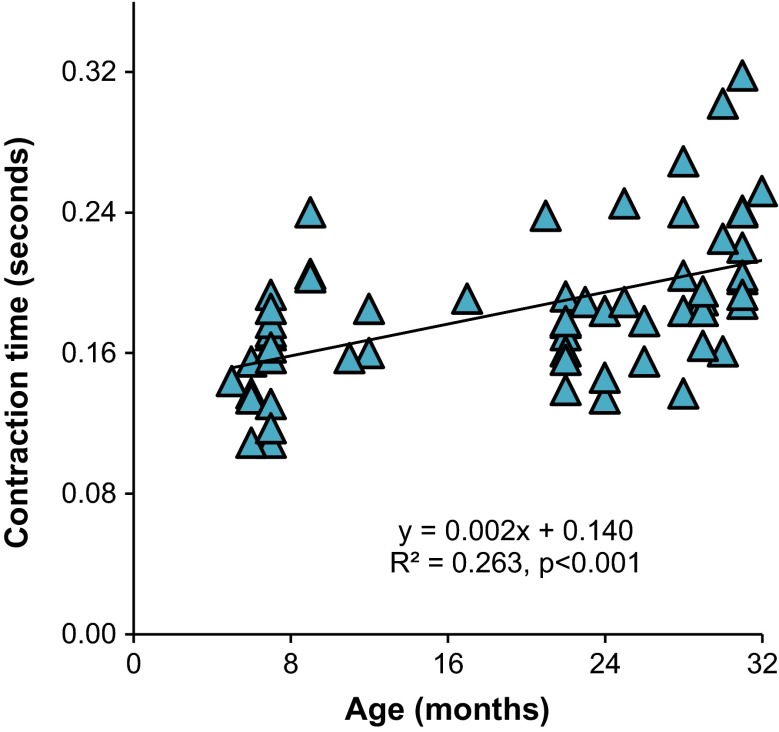
EDL contraction time to maximum force increases with age. Equation is a simple linear regression of time to maximum force in seconds with respect to age of the mouse in months. *Each symbol* represents the contraction time of one mouse

In the EDL, the time to reach maximum force (contraction time, measured at 150 Hz, average frequency for *P*_0_) was 0.159 ± 0.008, 0.178 ± 0.007, and 0.211 ± 0.010 s in adult, old, and elderly, respectively (Figure 6 shows regression in relation to age). There was no difference in the contraction time between the adult and old muscles, but the elderly had a contraction time 16 % longer than the old (*p* = 0.036) and 33 % longer than the adult (*p* < 0.001) (one-way ANOVA, Tukey’s HSD post hoc). Across the life span (5–32 months, *n* = 63), age had a modest correlation with EDL contraction time (*R* = 0.525, *p* < 0.001). In the SOL, there was no significant difference in contraction time (measured at 100 Hz).

## Discussion

The comprehensive analyses of power output and contractile velocity across the life span in both the EDL and SOL muscles provide novel insight into age-related contractile dysfunction. Overall, we found age-associated declines in power, velocity, and force in both the EDL and the SOL. The extent of decline varied with respect to % *P*_0_, as the velocity and power of both muscles were affected more by age when the muscles were contracting against the heavier loads (i.e., loads >50 % *P*_0_). The age-associated loss of both power and velocity at heavy loads has important functional implications, particularly in activities in which a large proportion of body mass must be moved. Getting up from a chair, acceleration of gait, and ascending or descending stairs are examples. Time to maximum force in the EDL increased with age, as did *a*/*P*_0_, a measure of the shape of the force-velocity curve. In addition, we found that decreases in velocity became a predictor of power loss as the load increased from light to heavy. Finally, in the EDL, with advancing age, there was a decline in the percentage of myosin light chain 3f (old < adult) and a decline in the relative MHC2b content.

There were *three* major motivating factors for this investigation. *First*, although maximal power generation occurs at 30–40 % *P*_0_, functional activities require movement that incorporates power generation across a wide load range, especially power generation under heavily loaded conditions. One critical heavily loaded condition occurs during fall prevention, where the rapid application of power to a limb is needed for an individual to regain balance. Other typical activities of daily living that require power generation at large percentages of maximum force are the opening of a stuck jar lid or rising to a standing position from a seat. Such activities of daily life are compromised in the older adult (Suzuki et al. 2001; Raj et al. 2010). Indeed, the loss of power has been suggested to be a strong predictor of disability (Puthoff and Nielsen 2007; Suzuki et al. 2001). Not only does the loss of power contribute to disability, but importantly, the velocity of the contraction itself correlates with physical performance and emphasizes the value of investigating this parameter under loaded conditions (Clemencon et al. 2008). *Second*, it is now well established in humans that resistance training with loads around 80 % of peak strength maximizes strength gains and is considered the best practice intervention for sarcopenia (Fiatarone et al. 1990; Evans 1998; Evans and Campbell 1993; Ali and Garcia 2014). *Third*, studying basic contractile properties over the entire load range and how they change over the life span is necessary because the information obtained provides a better perspective of the activities of daily living that are likely impacted, especially those requiring muscle force production >50 % *P*_0_ (i.e., heavy loads). In turn, this knowledge may lead to mechanistic investigations that provide targets pertinent to treatment strategies for sarcopenia that reverse frailty, increase power production, and improve quality of life in the elderly.

Interventions to prevent, reverse, or slow sarcopenia have mainly focused on strength gain and muscle hypertrophy. Based on the findings of this study, and assuming the results are translatable to humans, designing training programs for muscle power and velocity gains at higher percentages of maximum force would meaningfully impact performance for the older adult. Previous reports show greater improvements in power production when training for velocity of contraction, power training, compared to training for strength (Earles et al. 2001). More importantly, functional ability is improved greatly with power training. For example, power training in community-dwelling older adults was shown to have a greater impact than strength training on functional ability, such as measured by the Continuous Scale Physical Functional Performance test (Miszko et al. 2003). Thus, some combination of exercise training strategies that include strength/hypertrophy, as well as power and velocity training, may be required to maximize functional improvement and to mitigate sarcopenia/frailty. This combined exercise training strategy will likely require additional support through ergogenic and nutritional supplementation to assist in overcoming the anabolic resistance reported in the elderly and to maximize gains, since anabolic resistance has a multifactorial etiology (Haran 2012).

### Peak values—*P*_max_, *V*_max_, and *P*_0_

Previous studies report the traditionally measured peak values of power (*P*_max_), velocity (*V*_max_), and force (*P*_0_). With age, both *P*_max_ and *P*_0_ decline (Lynch et al. 2001; Narici et al. 2008; Brooks and Faulkner 1988a, b; Graber et al. 2013; Phillips et al. 1991), whereas *V*_max_ (derived from the force-velocity curve) is relatively unaffected (Brooks and Faulkner 1988a; Lynch et al. 2001). Peak values in the current study are consistent with these results.

Our study found key differences in two contractility outcome measures, *a*/*P*_0_ and contractile velocities over the load range (at both low and high loads). Our comprehensive analysis revealed that, with age, there were significant increases in the *a*/*P*_0_ ratio, suggesting a change in the force-velocity relationship (Jones 2010), as well as decreases in power production and contractile velocity. These differences likely are attributed to the age of the animals and to differences in the experimental design for evaluation of the force-velocity and force-power curves. Our oldest group of mice was 2–3 months older (mean age 30 months) than the oldest mice in previous studies (mean ages 27–28 months). In addition, previous studies only measured velocity at percentages of *P*_0_ less than 50 %.

The current study reports other novel findings: the impact of age on both velocity and power during heavily loaded contractions, the increased relationship (*R* value) between velocity and power at larger percentages of *P*_0_, the reduced percentage of maximum force at which maximum power was produced in the EDL as a function of age, the increased contraction time to maximum force in the EDL, and the comprehensive characterization of the decline in velocity and power over the life span (5–32 months) of C57BL/6 mice via linear regression.

### Mechanisms underlying age-related power impairment

Any reduction in force or velocity will result in loss of power. The loss of force production is generally accepted to be associated with muscle atrophy, motor unit loss, and deficits in muscle quality (Raj et al. 2010). Changes in connective tissue, such as a decrease in elastic modulus in tendons (Onambele et al. 2006), and alterations in muscle architecture, such as pennation angle, are also likely to influence contractility negatively (Thom et al. 2007) by diminishing force transmission. However, the reduction in force capacity does not entirely explain the accelerated loss of power seen with age (Thom et al. 2005).

Our findings that the *a*/*P*_0_ ratio increased with age also suggest a change in the force-velocity relationship (Jones 2010). Age-related reduction in contraction velocity may also contribute to loss of power. In the human and rodent literature spanning various experimental technologies, such as single skeletal muscle fibers and applied human performance, an age-related loss of velocity has been reported (Thompson and Brown 1999; Kim et al. 2012; Li and Larsson 1996; Krivickas et al. 2001; Larsson et al. 1997; Narici et al. 1991; Thom et al. 2007), with some disagreement in studies of human single muscle fibers (Trappe et al. 2003). In numerous in vivo human studies, many potential contributors to loss of velocity and power have been suggested: neurological changes; motor unit recruitment (Narici et al. 2008); increased proportion of fat/connective tissue in older muscle (Addison et al. 2014); muscle architecture changes, including pennation angle and fiber fascicle length reductions (Klein et al. 2001); and deleterious joint/range of motion/mobility changes (Lanza et al. 2003).

Likely candidates for the age-related velocity decline within individual skeletal muscle fibers include impaired actin/myosin interactions (Höök et al. 2001; Raj et al. 2010) and changes in myosin heavy and light chain isoform proportions (Moss et al. 1995; Larsson et al. 1997). The main factor that determines the velocity of a muscle cell contraction is the MHC isoform proportion/distribution of the fiber (Schiaffino et al. 1989). We found a small but significant age-associated shift in MHC content in the EDL with a 6 % decline in MHC2b in the elderly mice that might explain some of the velocity change. Contractile velocity of a single fiber is also regulated both by the phosphorylation state of the regulatory MLC2 and the isoform composition of the essential light chains (Grange et al. 1995), with MLC3f being the fastest (Kim et al. 2012). We found a 21 % decline in MLC3f relative content in EDL fibers that, together with the decrease in MHC2b, might partially explain the reduced velocity. In previous work from our laboratory, single fibers from rat semimembranosus showed a 69 % decline in %MLC3f content in MHC type II fibers with age. However, upregulation of MLC3f using gene therapy restored single-fiber-unloaded contractile velocity in older rats to adult levels (Kim et al. 2012). Further investigation is needed to clarify the roles of myosin isoform changes and their contributions to velocity and power loss.

### Possible mechanisms of increased age-associated dysfunction at higher loads

At low loads, the muscle function was better preserved; however, at high loads, the impairments in myosin-actin interactions were exacerbated leading to reduced contractility. Indeed, myosin working stroke distance and velocity are reduced at higher loads (Reconditi et al. 2004). We believe that age-associated changes in actin-myosin cross-bridge kinetics, such as the ratio of strong and weak structural states, which result in fewer strong binding attachments, decreased detachment rates, and increased internal drag, play a role in load-associated decline of function with age (Prochniewicz et al. 2005). Further investigation is needed.

Our finding of an age-related increase in EDL contraction time to maximum force suggests connective tissue dysfunction as an underlying cause of power impairment (Narici et al. 2008). At the tissue level, the tendons, myotendinous junction, and other connective tissues responsible for force transmission have reduced functionality with age (Onambele et al. 2006; Zhang and Gao 2014). These age-related changes result in thickening of the extracellular matrix and detrimental mechanical alterations to the tendon, caused by a decrease in elastic modulus and stiffness resulting in increased compliance, which negatively alter force transmission speed and ultimately contractile velocity (McCarthy and Hannafin 2014; LaCroix et al. 2013; Narici et al. 2008). We propose that these detrimental modifications in connective tissue are manifested at a greater extent and contribute to increased dysfunction when the muscle is contracting against higher load. Further investigation is needed to delineate the contribution of connective tissue decline to loss of contractile efficiency.

It is important to note that lifestyle and behavior may play a role in dysfunctional power production at heavy loads. As organisms age, not only does the general rate of activity decline, but the tendency to engage in behaviors that require near maximal exertion declines as well (Jefferis et al. 2014). For example, muscles used for postural activities often have less age-related decline than muscles designed for explosive movements (SOL vs. EDL in the current study, for example). In addition, motor unit reorganization with age does not favor the maintenance of fast-twitch anaerobic activities, due to preferential type 2 motor unit net loss and the more general preservation of type 1 myosin muscle fibers with age (Luff 1998; Thompson et al. 2006). A higher proportion of type 2 fibers allows for greater power and torque generation (Fitts and Widrick 1996). Thus, the disproportionate loss of type 2 motor units with age likely contributes to loss of power and contractile velocity (Faulkner et al. 2007). Denervation and subsequent reinnervation are balanced in youth, but become unbalanced in favor of denervation, atrophy, and a net loss of fibers in older adults (Koopman and van Loon 2009; Breen and Phillips 2011; Ryall et al. 2008). For instance, between age 50 and 80, approximately 50 % of muscle cells are lost (Faulkner et al. 2007).

## Conclusion

There was an age-associated decline in power, velocity, and force in both the EDL and SOL muscles of the C57BL/6 male mouse. Specifically, contractions at heavy compared to light loads showed an exacerbated age-associated decline of power and velocity. The shape of the force-velocity curve was also altered with age, with *a*/*P*_0_ increasing. We also detected a reduction in EDL MLC3f and MHC2b content, which may contribute to the declining velocity of contraction. Our finding showing an age-related increase in time to maximum force in the EDL suggests that, among other potential mechanisms such as impaired calcium handling, connective tissue dysfunction may play a role in power and velocity loss. Further investigation is needed to determine the specific mechanisms underlying age-associated increased dysfunction in muscles contracting concentrically under heavily loaded conditions.

## Electronic supplementary material

Online Resource 1 (PDF 89 kb)

Online Resource 2 (PDF 704 kb)

## References

[CR1] Addison O, Marcus RL, Lastayo PC, Ryan AS (2014). Intermuscular fat: a review of the consequences and causes. Int J Endocrinol.

[CR2] Ali S, Garcia JM (2014). Sarcopenia, cachexia and aging: diagnosis, mechanisms and therapeutic options—a mini-review. Gerontology.

[CR3] Breen L, Phillips S (2011). Skeletal muscle protein metabolism in the elderly: interventions to counteract the ‘anabolic resistance’ of ageing. Nutr Metab.

[CR4] Brooks SV, Faulkner JA (1988). Contractile properties of skeletal muscles from young, adult and aged mice. J Physiol.

[CR5] Brooks SV, Faulkner JA (1988). Maximum and sustained power of extensor digitorum longus muscles from young, adult, and old mice. J Gerontol.

[CR6] Burkholder TJ, Fingado B, Baron S, Leiber RL (1994). Relationship between muscle fiber types and sizes and muscle architectural properties in the mouse hindlimb. J Morphol.

[CR7] Chodzko-Zajko W, Proctor DN, Fiaterone Sing MA, Minson CT, Nigg CR, Salem GL, Skinner JS (2009). American College of Sports Medicine position stand. Exercise and physical activity for older adults. Med Sci Sports Exerc.

[CR8] Clemencon M, Hautier CA, Rahmani A, Cornu C, Bonnefoy M (2008). Potential role of optimal velocity as a qualitative factor of physical functional performance in women aged 72 to 96 years. Arch Phys Med Rehabil.

[CR9] Dalton BH, Allen MD, Power GA, Vandervoort AA, Rice CL (2014). The effect of knee joint angle on plantar flexor power in young and old men. Exp Gerontol.

[CR10] Earles DR, Judge JO, Gunnarsson OT (2001). Velocity training induces power-specific adaptations in highly functioning older adults. Arch Phys Med Rehabil.

[CR11] Evans WJ, Campbell WW (1993). Sarcopenia and age-related changes in body composition and functional capacity. J Nutr.

[CR12] Evans WJ (1998). Exercise training guidelines for the elderly. Med Sci Sports Exerc.

[CR13] Faulkner J, Larkin L, Claflin D, Brooks S (2007). Age-related changes in the structure and function of skeletal muscles. Clin Exp Pharmacol Physiol.

[CR14] Fiatarone MA, Marks EC, Ryan ND, Meredith CN, Lisitz LA, Evans WJ (1990). High-intensity strength training in nonagenarians. Effects on skeletal muscle. JAMA: J Am Med Assoc.

[CR15] Fielding RA, Vellas B, Evans WJ, Bhasin S, Morley JE, Newman AB, Abellan van Kan G, Andrieu S, Bauer J, Breuille D, Cederholm T, Chandler J, De Meynard C, Donini L, Harris T, Kannt A, Keirne-Guibert F, Onder G, Papanicolaou D, Rolland Y, Rooks D, Sieber C, Souhami E, Verlaan S, Zamboni M (2011). Sarcopenia: an undiagnosed condition in older adults. Current consensus definition: prevalence, etiology, and consequences. International Working Group on Sarcopenia. JAMA: J Am Med Dir Assoc.

[CR16] Fitts RH, Widrick JJ (1996). Muscle mechanics: adaptations with exercise-training. Exerc Sport Sci Rev.

[CR17] Graber TG, Ferguson Stegall L, Kim J, Thompson LV (2013). C57Bl/6 neuromuscular healthspan scoring system. J Gerontol A Biol Sci Med Sci.

[CR18] Grange RW, Cory CR, Vandenboom R, Houston ME (1995). Myosin phosphorylation augments force-displacement and force-velocity relationships of mouse fast muscle. Am J Physiol.

[CR19] Haran PH (2012). Role and potential mechanisms of anabolic resistance in sarcopenia. J Cachex Sarcopenia Muscle.

[CR20] Höök P, Sriramoju V, Larsson L (2001). The effect of aging on actin sliding speed on myosin from single skeletal muscle cells of mice, rats and humans. Am J Physiol.

[CR21] Jefferis BJ, Sartini C, Ash S, Lennon LT, Wannmethee SG, Lee IM, Whincup PH (2014). Trajectories of objectively measured physical activity in free-living older men. Med Sci Sports Exerc.

[CR22] Jones DA (2010). Changes in the force-velocity relationship of fatigued muscle: implications for power production and possible causes. J Physiol.

[CR23] Kim J, Torgerud WS, Mosser KH, Hirai H, Watanabe S, Asakura A, Thompson LV (2012). Myosin light chain 3f attenuates age-induced decline in contractile velocity in MHC type II single muscle fibers. Aging Cell.

[CR24] Klein CS, Rice CL, Marsh GD (2001). Normalized force, activation, and coactivation in the arm muscles of young and old men. J Appl Physiol.

[CR25] Koopman R, van Loon L (2009). Aging, exercise, and muscle protein metabolism. J Appl Physiol.

[CR26] Krivickas LS, Suh D, Wilkims J, Va H, Roubenoff R, Frontera WR (2001). Age- and gender-related differences in maximum shortening velocity of skeletal muscle fibers. Am J Phys Med Rehab.

[CR27] LaCroix AS, Duenwald-Kuehl SE, Brickson S, Akins TL, Diffee G, Aiken J, Vanderby R, Lakes RS (2013). Effect of age and exercise on the viscoelastic properties of rat tail tendon. Ann Biomed Eng.

[CR28] Landi F, Cruz-Jentft AJ, Liperti R, Russo A, Giovannini S, Tosato M, Capoluongo E, Bernabei R, Onder G (2013). Sarcopenia and mortality risk in frail older persons aged 80 years and older: results from ilSIRENTE study. Age Ageing.

[CR29] Lanza IR, Towse GE, Caldwell DM, Wigmore DM, Kent-Braun JA (2003). Effects of age on human muscle torque, velocity and power in two muscle groups. J Appl Physiol.

[CR30] Larsson L, Li X, Frontera WR (1997). Effects of aging on shortening velocity and myosin isoform composition in single human skeletal muscle cells. Am J Physiol.

[CR31] Li X, Larsson L (1996). Maximum shortening velocity and myosin isoforms in single muscle fibers from young and old rats. Am J Physiol.

[CR32] Luff AR (1998). Age-associated changes in the innervation of muscle fibers and changes in the mechanical properties of motor units. Ann N Y Acad Sci.

[CR33] Lynch GS, Hinkle RT, Chamberlain JS, Brooks SV, Faulkner JA (2001). Force and power output of fast and slow skeletal muscles from mdx mice 6–28 months old. J Physiol.

[CR34] Macklai NS, Spagnoli J, Junod J, Santos-Eggimann B (2013). Prospective association of the share-operationalized frailty phenotype with adverse health outcomes: evidence from 60+ community-dwelling Europeans living in 11 countries. BMC Geriatr.

[CR35] McCarthy M, Hannafin JA (2014). The mature athlete: aging tendon and ligament. Sports Health.

[CR36] Mendez J, Keys A (1960). Density and composition of mammalian muscle. Metabolism.

[CR37] Miszko TA, Cress E, Slade J, Covey CJ, Agrawal SK, Doerr CE (2003). Effect of strength and power training on physical function in community-dwelling older adults. J Gerontol A: Biol Med Sci.

[CR38] Moss RL, Diffee GM, Greaser ML (1995). Contractile properties of skeletal muscle fibers in relation to myofibrillar protein isoforms. Rev Physiol Biochem Pharmacol.

[CR39] Narici MV, Maffulli N, Maganaris CN (2008). Ageing of human muscles and tendons. Disabil Rehabil.

[CR40] Narici MV, Bordini M, Cerretelli P (1991). Effect of aging on human adductor pollicis muscle function. J Appl Physiol.

[CR41] Onambele GL, Narici MV, Maganaris CN (2006). Calf muscle-tendon properties and postural balance in old age. J Appl Physiol.

[CR42] Phillips SK, Bruce SA, Woledge RC (1991). In mice, the muscle weakness due to age is absent during stretching. J Physiol.

[CR43] Power GA, Dalton BH, Rice CL (2013). Human neuromuscular structure and function in old age: a brief review. Journal of Sport and Health Science.

[CR44] Prochniewicz E, Thomas D, Thompson LV (2005). Age-related decline in actomyosin function. J Gerontol A Biol Sci Med Sci.

[CR45] Puthoff ML, Nielsen DH (2007). Relationships among impairments in lower-extremity strength and power, functional limitations, and disability in older adults. Phys Ther.

[CR46] Raj IS, Bird SR, Shield AJ (2010). Aging and the force-velocity relationship of muscles. Exp Gerontol.

[CR47] Reconditi M, Linari M, Lucii L, Stewart A, Sun YB, Boesecke P, Narayanan T, Fischetti RF, Irving T, Piazzesi G, Irving M, Lombardi V (2004). The myosin motor in muscle generates a smaller and slower working stroke at higher load. Nature.

[CR48] Reid KF, Fielding RA (2012). Skeletal muscle power: a critical determinant of physical functioning in older adults. Exerc Sport Sci Rev.

[CR49] Reid KF, Pasha E, Doros G, Clark DJ, Patten C, Phillips EM, Frontera WR, Fielding RA (2014). Longitudinal decline of lower extremity muscle power in healthy and mobility-limited older adults: Influence of muscle mass, strength, composition, neuromuscular activation and single fiber contractile properties. Eur J Appl Physiol.

[CR50] Ryall J, Schertzer J, Lynch G (2008). Cellular and molecular mechanisms underlying age-related skeletal muscle wasting and weakness. Biogerontology.

[CR51] Schiaffino S, Gorza L, Sartore S, Saggin L, Ausoni S, Vianello M, Gunderson K, Lorno T (1989). Three myosin heavy chain isoforms in type 2 skeletal muscle fibres. J Muscle Res Cell Motil.

[CR52] Suzuki T, Bean JF, Fielding RA (2001). Muscle power of the ankle flexors predicts functional performance in community-dwelling older women. J Am Geriatr Soc.

[CR53] Thom JM, Morse CI, Birch KM, Narici MV (2005). Triceps surae muscle power, volume, and quality in older versus younger healthy men. J Gerontol A: Biol Med Sci.

[CR54] Thom JM, Morse CI, Birch KM, Narici MV (2007). Influence of muscle architecture on the torque and power-velocity characteristics of young and elderly men. Eur J Appl Physiol.

[CR55] Thompson LV, Brown M (1999). Age-related changes in contractile properties of single skeletal fibers from the soleus muscle. J Appl Physiol.

[CR56] Thompson LV, Durand D, Fugere NA, Ferrington DA (2006). Myosin and actin expression and oxidation in aging muscle. J Appl Physiol.

[CR57] Thompson LV (2009). Age-related muscle dysfunction. Exp Gerontol.

[CR58] Trappe S, Gallagher P, Harber M, Carrithers J, Fluckey J, Trappe T (2003). Single muscle fibre contractile properties in young and old men and women. J Physiol.

[CR59] Woledge RC, Curtin NA, Homsher E (1985). Energetic aspects of muscle contraction. [Monographs of the Physiological Society; no. 41].

[CR60] Zhang C, Gao Y (2014). Effects of aging on the lateral transmission of force in rat skeletal muscle. J Biomech.

