# Supplementary material for: C57BL/6 life span study: age-related declines in muscle power production and contractile velocity
Source: Age (Dordr). 2015 Apr 17;37(3):36. doi: 10.1007/s11357-015-9773-1 (PMC4401475; doi:10.1007/s11357-015-9773-1)
Supplement: Supplementary file 1 — (PDF 89 kb) [file 11357_2015_9773_MOESM1_ESM.pdf]

Online Resource 1
APPENDIX: Relationships between Force, Velocity, and Maximum Power

In order to evaluate the relationship between velocity, force and maximum power, we examined simple and multiple linear regressions to determine how much variability is explained by each component of power [P_0, velocity, and incorporating $\%P_0@P_{max}$ (the percentage of maximum force where the muscle produced maximum power) and age to hierarchical multiple regression analysis]. In the EDL, P_0 explained 58% of the individual variability in P_{max} (P_0 + age, 66%). At 30%P_0 the velocity of contraction explained 23% of P_{max} (velocity + age, 49%), and when the two components (P_0 and velocity at 30% P_0) were combined 73% of P_{max} variability was explained. Adding a third component, age, 75% of the variability in P_{max} was explained. In the SOL, the P_0 explains 59% of the individual variability (P_0 + age, not significantly different). At 20%P_0 the velocity of contraction explains 50% of the variability (velocity + age, no difference), and when combining the two components, 83% is explained (adding age yields 84% of the variability). (**Online Resource 2**, **Figure S5**)

For simplicities sake, we will use the convention of V30 to represent velocity (v) as measured at 30% of P_0 (30). Simple linear regression demonstrated a strong positive correlation, as would be expected, of EDL P_{max} with P_0 [R=0.79, r^2=0.630, p-value<.001; equation: Pmax (mN*fl/s) = -33.48 + 1.36 * P_0]; as well as with contractile velocity at 30%P_0 [R=0.51, r^2=0.260, p<.001; equation: P_{max} (mN*fl/s) = -0.90 + 102.43 * v30]. (**Online Resource 2, Figure S5**) We performed a factor analysis (promax rotation) to determine which of the collected outcome variables contributed most to the variability in the system (overall model). The results of our factor analysis yielded that P_0, v30, v40 and v60 were the main sources of variability in P_{max}. Interestingly, the only velocity that was not significantly correlated with the EDL P_{max} was V_{max}.

After analyzing the velocities separately (**Online Resource 2, Figure S1**), v30 was selected to be used in the multiple regression analysis and the combination of P_0 and v30 explained 79% of the variability in the observed P_{max} (R=0.853, r^2=0.728, p<.001; equation: P_{max} (mN*fl/s) = -316.33 + 1.22 * P_0 + 77.8 * v30]). %P_0@P_{max} was correlated with P_{max} (R=0.398, r^2=0.159, p=.001), but did not significantly add information when combined with the above multiple regression (R=0.891). When age was incorporated into the model (multiple regression of P_{max} with P_0, V30 and age of mouse); however, the r^2 was increased to 0.749 (coefficient significance p=.032). %P_0@P_{max} was correlated with P_{max} (R=0.398, r^2=0.159, p=0.001), but did not significantly add information when combined to the multiple regression.

P_{max} in the SOL followed a similar pattern. Regression of P_{max} with P_0 (R=0.762, r2=.577, p<.001; equation: P_{max} (mN*fl/s) = -17.65 + 0.46 * P_0]; as well as with contractile velocity at v20 [R=0.714, r^2=0.501, p<.001; equation: P_{max} (mN*fl/s) = -13.78 + 46.78 * v20] showed strong correlations. A principle component factor analysis revealed that the top 4 factors, in descending order of R-values (in parenthesis), correlating to the P_{max} in the SOL were: P_0 (0.762), v20 (0.714), V_{max} (0.71) and v30 (0.637). The most explanatory/predictive equation was derived from a regression of P_{max} with P_0 and v20 [R=0.915, r^2=0.837, p<.001; equation: P_{max} (mN*fl/s) = -58.412 + 0.359 * P_0 + 34.842 * v30]. In the SOL, the %P_0@P_{max} was not significantly correlated with P_{max}.
