# Supplementary material for: C57BL/6 life span study: age-related declines in muscle power production and contractile velocity
Source: Age (Dordr). 2015 Apr 17;37(3):36. doi: 10.1007/s11357-015-9773-1 (PMC4401475; doi:10.1007/s11357-015-9773-1)
Supplement: Supplementary file 2 — (PDF 704 kb) [file 11357_2015_9773_MOESM2_ESM.pdf]

Online Resource 2
Supplemental Figures S1-S7

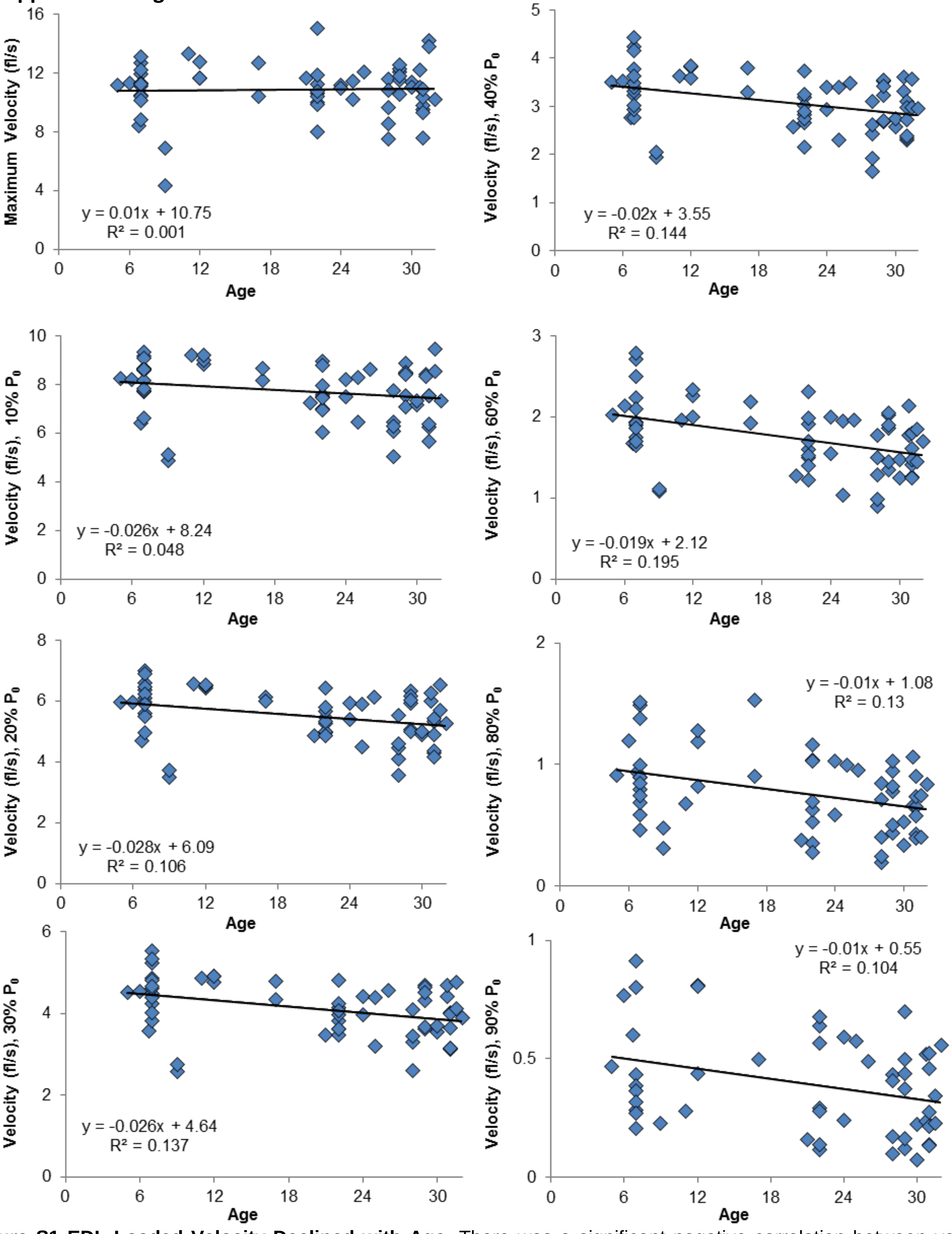

Figure S1 EDL Loaded Velocity Declined with Age. There was a significant negative correlation between velocity and age at the measured loads (20-90%P_0). V_{max}, however, (which was non-physiological and represented what velocity would be if a muscle was completely unloaded) did not change in a linear manner over the lifespan. Each diamond represents a measurement from an individual mouse. Age: Age of mouse in months. Equation: simple linear regression of velocity (y) as a function of age (x). fl/s = fiber lengths per second. x% P_0 = percentage of maximum tetanic force when the velocity measurement was taken. Maximum Velocity: V_{max}, maximum unloaded velocity.

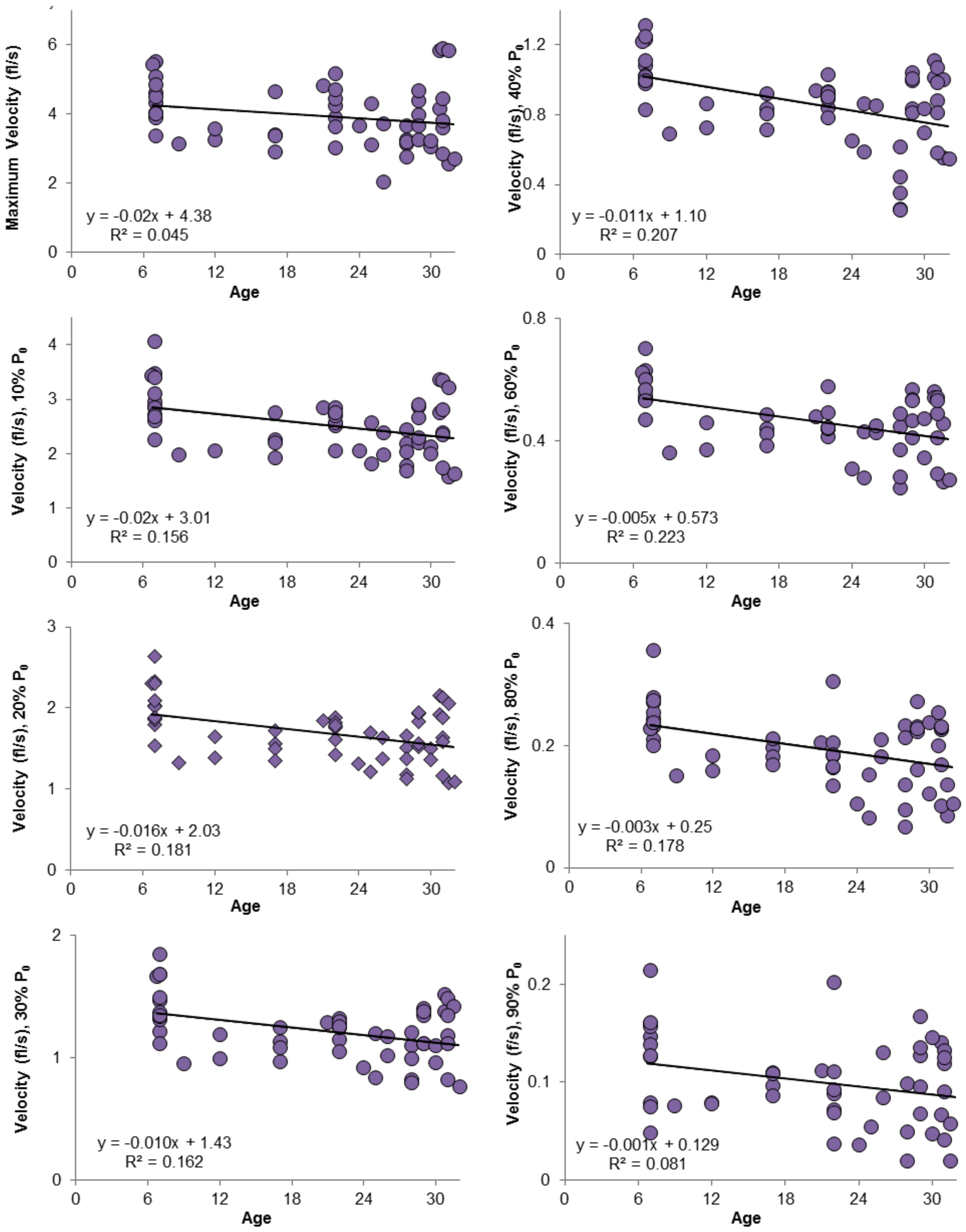

Figure S2 SOL Loaded Velocity Declined with Age. There was a significant negative correlation between velocity and age at the measured loads (10-90%P_0). V_{max} (which was non-physiological and represented what velocity would be if a muscle was completely unloaded) did not change in a significant linear manner over the lifespan. Each diamond represents a measurement from an individual mouse. Age: Age of mouse in months. Equation: simple linear regression of velocity (y) as a function of age (x). fl/s = fiber lengths per second. x% P_0 = percentage of maximum tetanic force when the velocity measurement was taken. Maximum Velocity: V_{max}, maximum unloaded velocity.

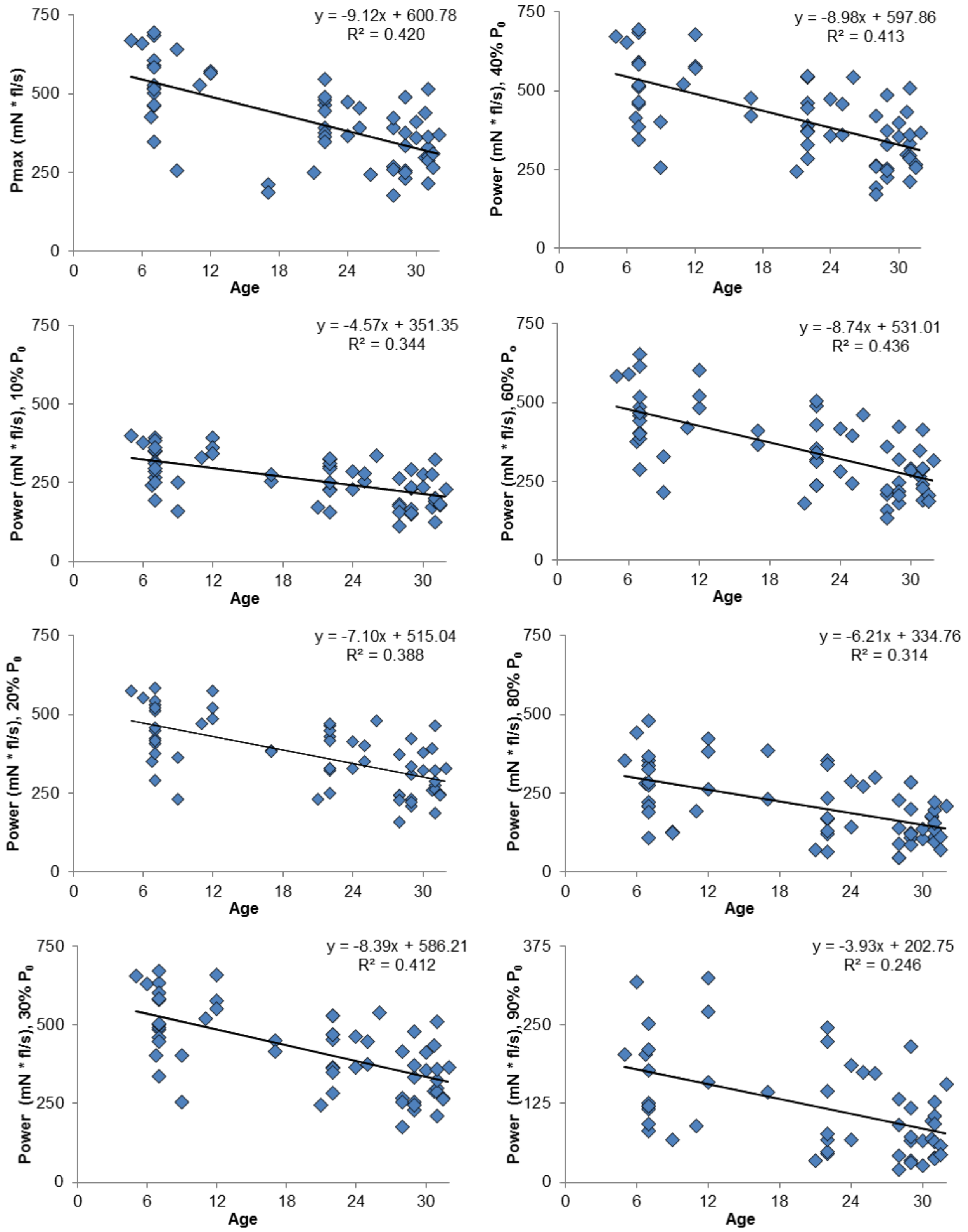

Figure S3 EDL Power Declined with Age. There was a significant negative linear correlation between power production and age at the measured loads (10-90%P_0) and at P_{max} (maximum power). x% P_0= "x" percentage of maximum tetanic force when the power measurement was derived. Each symbol (diamond) represents a measurement from an individual mouse. Power measured in milliNewtons * fiber lengths per second (mN * fl/s). Age: age of mouse in months. Equation: simple linear regression of power (y) as a function of age (x).

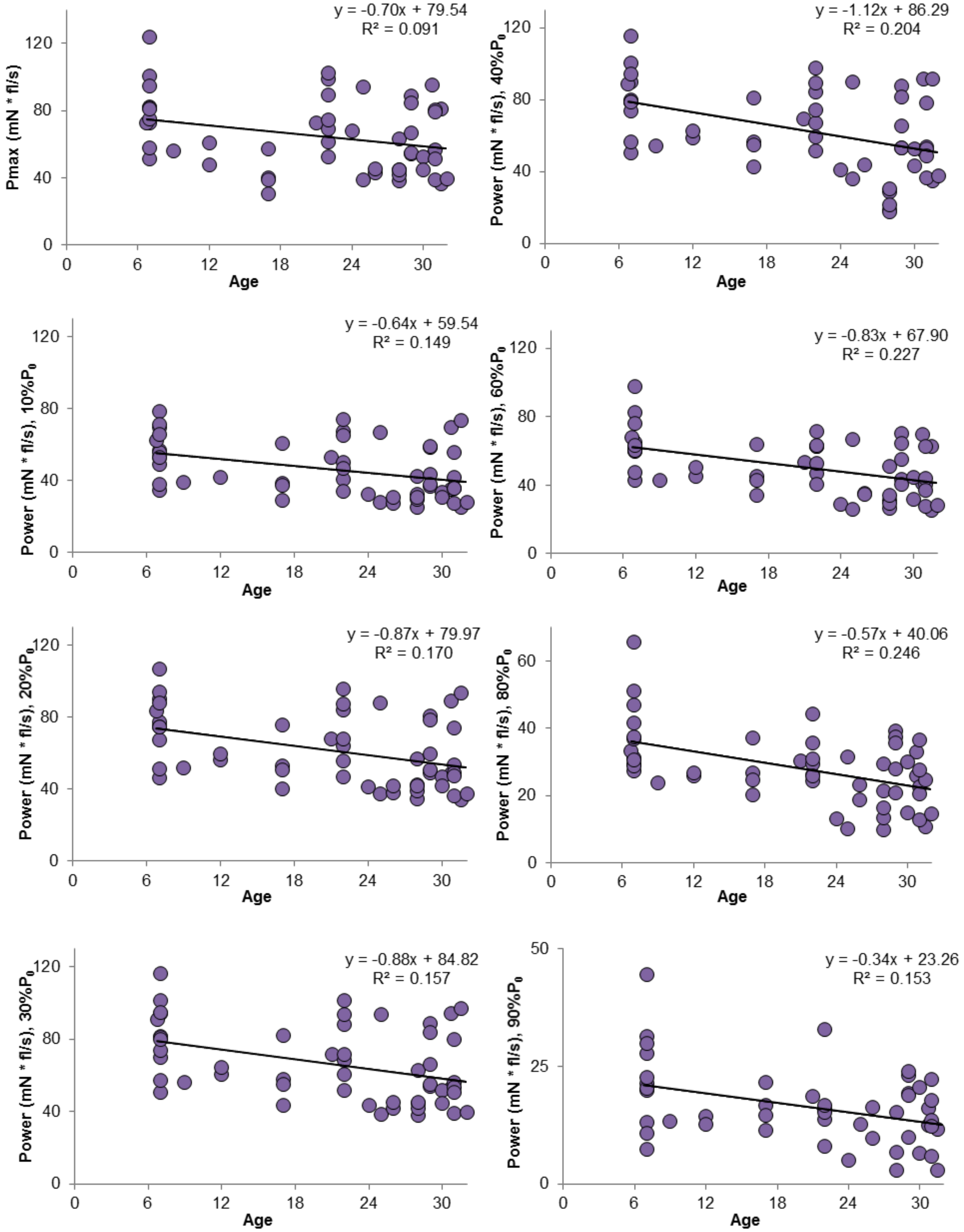

Figure S4 SOL Power Production Declined with Age. There was a significant negative correlation between power and age at the measured loads (10-90%P_0) and at P_{max}. Power measured in milliNewtons * fiber lengths per sec (mN*fl/sec). x% P_0= "x" percentage of maximum tetanic force when the power measurement was derived. Each symbol (circle) represents a measurement from an individual mouse. Age: age of mouse in months. Equation: simple linear regression of power (y) as a function of age (x).

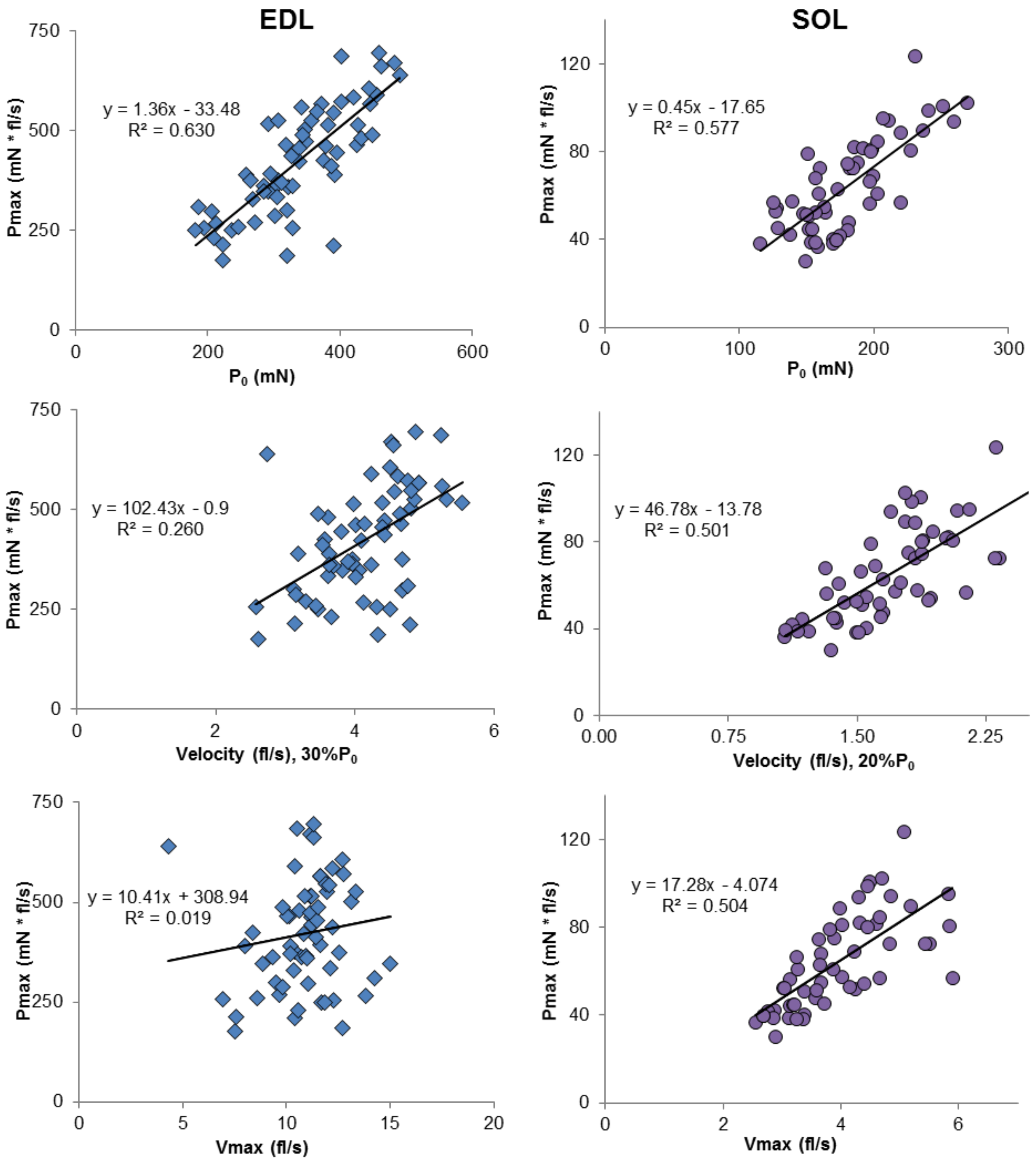

Figure S5 Relationship of Pmax, P_0 and Velocity. In both the EDL and SOL, P_{max} (maximum power) and P_0 (maximum tetanic force) (R=0.79 and 0.76, respectively, both p<0.001), and P_{max} and Velocity (R=0.51 and 0.71, both p<0.001) were correlated. In the EDL, P_{max} and V_{max} (maximum unloaded velocity) were not related. However, there was a significant correlation (R=0.71) between SOL V_{max} and P_{max}. P_{max} measured in milliNewtons * fiber lengths per second (mN * fl/s) and velocity measured in fiber lengths per second (fl/s). Each symbol (diamond for EDL and circle for SOL) represents measurements taken from one mouse. Equation is of a simple linear regression of P_{max} (y) being a function of the x-axis variable (P_0, velocity x% of P_0, or V_{max}).

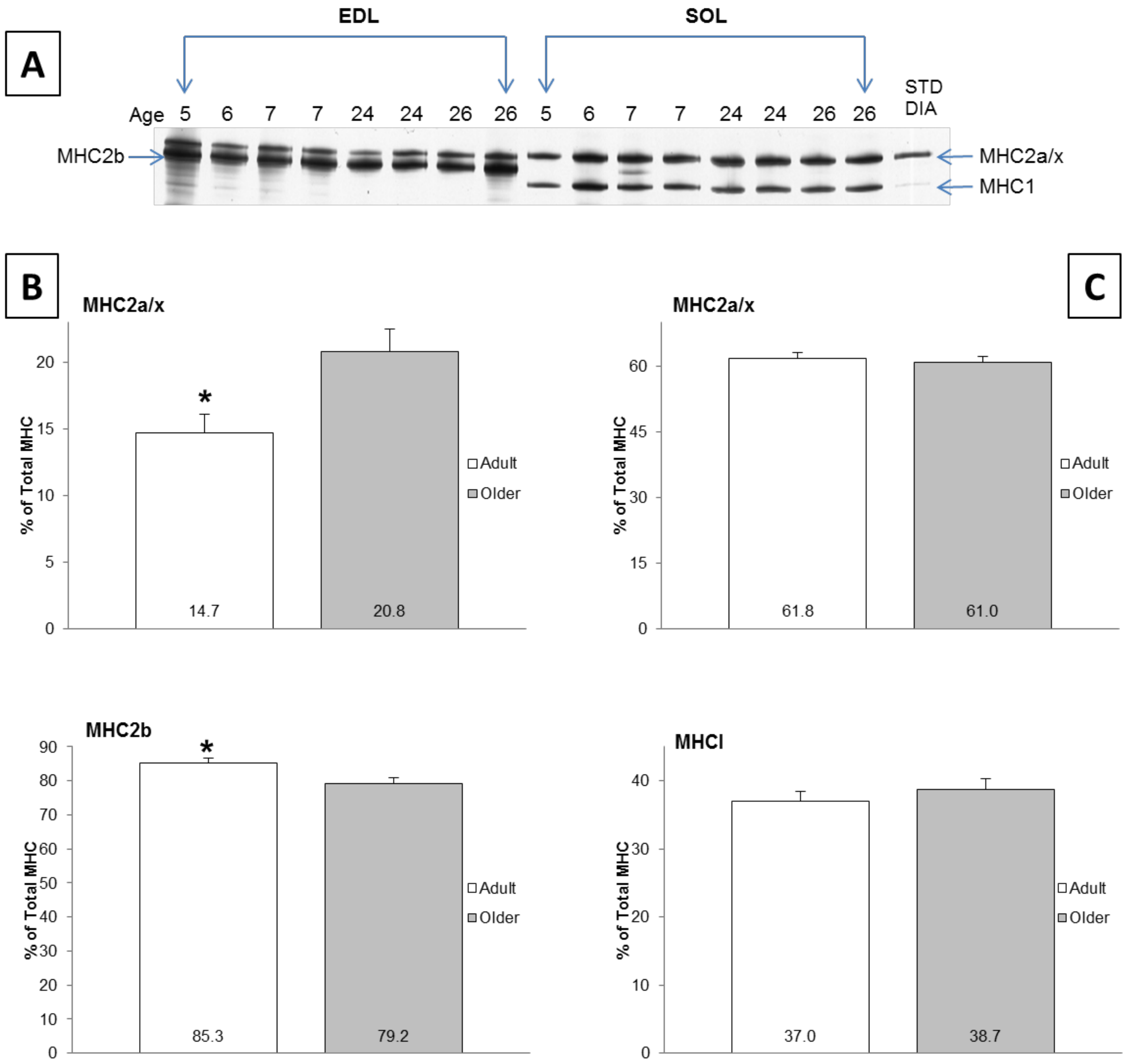

Figure S6 MHC Composition with Age. A. Representative Gel Image. Silverstained 5% Acrylamide large format gel. **B. EDL** Expression of MHC2/ax increased by 6% in older (21%, n=25, mean age=28 months, p=0.009) compared to adult mice (15%, n=18, mean age=7 months) using Student's t-test, implying a shift towards a slower isoform composition. Conversely, MHC2b expression is reduced by 6% with age (Adult 85%, Old 79%, p=0.01). **C. SOL** There was no change in the SOL MHC expression profile (Adult: MHC2ax 62%, MHC1 37%, n=15, mean age=7.8 months; Older: MHC2ax 61%, MHC1 39%, n=18, mean age=27.8). Each lane represents whole homogenized muscle of one mouse at the age listed on top. Age: age in months of mouse. STD DIA: standard from rat diaphragm (proteins identified using mass spectrometry).

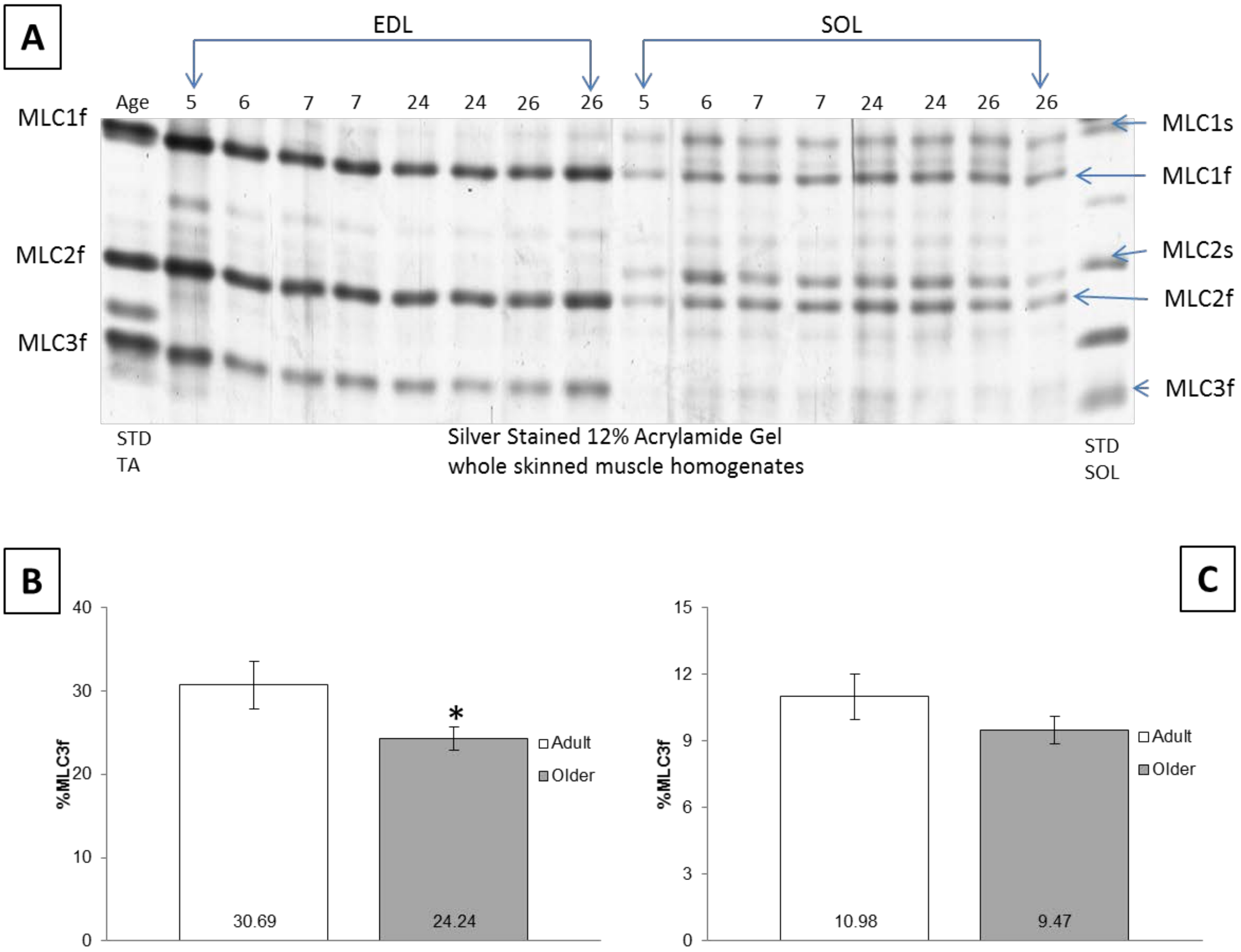

Figure S7 MLC3f Percentage Decreased 21% with Age in EDL. A. Representative Gel Image. Silver-stained 12% Acrylamide large format gel. **B. EDL** There was a 21% decline in MLC3f with age**. C. SOL** There was no change in MLC3f. Adult group (EDL, 31% MLC3f, n=19, mean age=7.5; SOL % MLC3f, n=18, mean age=7.4) was compared to combined old/elderly group, Older (EDL, 24% MLC3f, n=23, mean age =27.9; SOL, % MLC3f, n=22, mean age=27.8) using Student's t-test (EDL, p=0.03; SOL p=0.20). Each lane represents whole homogenized permeabalized muscle of one mouse at the age listed on top. Age= age in months of mouse. STD TA: rat tibialis anterior standard. STD SOL: rat SOL standard. Standard proteins identified via mass spectrometry. MLC (myosin light chain) is shown with three essential light chain isoforms (1s, 1f, 3f) and 2 two regulatory light chain isoforms (2s, 2f). s = slow. f = fast .Number in base of columns in chart equals average percentage of MLC3f out of total. Note: %MLC3f is out of the total of fast MLC [MLC3f / (MLC1f+MLC3f)], in SOL MLC1s was not counted.
